# Association between pre-biologic T2-biomarker combinations and response to biologics in patients with severe asthma

**DOI:** 10.3389/fimmu.2024.1361891

**Published:** 2024-04-19

**Authors:** Celeste M. Porsbjerg, John Townend, Celine Bergeron, George C. Christoff, Gregory P. Katsoulotos, Désirée Larenas-Linnemann, Trung N. Tran, Riyad Al-Lehebi, Sinthia Z. Bosnic-Anticevich, John Busby, Mark Hew, Konstantinos Kostikas, Nikolaos G. Papadopoulos, Paul E. Pfeffer, Todor A. Popov, Chin Kook Rhee, Mohsen Sadatsafavi, Ming-Ju Tsai, Charlotte Suppli Ulrik, Mona Al-Ahmad, Alan Altraja, Aaron Beastall, Lakmini Bulathsinhala, Victoria Carter, Borja G. Cosio, Kirsty Fletton, Susanne Hansen, Liam G. Heaney, Richard B. Hubbard, Piotr Kuna, Ruth B. Murray, Tatsuya Nagano, Laura Pini, Diana Jimena Cano Rosales, Florence Schleich, Michael E. Wechsler, Rita Amaral, Arnaud Bourdin, Guy G. Brusselle, Wenjia Chen, Li Ping Chung, Eve Denton, Joao A. Fonseca, Flavia Hoyte, David J. Jackson, Rohit Katial, Bruce J. Kirenga, Mariko Siyue Koh, Agnieszka Ławkiedraj, Lauri Lehtimäki, Mei Fong Liew, Bassam Mahboub, Neil Martin, Andrew N. Menzies-Gow, Pee Hwee Pang, Andriana I. Papaioannou, Pujan H. Patel, Luis Perez-De-Llano, Matthew J. Peters, Luisa Ricciardi, Bellanid Rodríguez-Cáceres, Ivan Solarte, Tunn Ren Tay, Carlos A. Torres-Duque, Eileen Wang, Martina Zappa, John Abisheganaden, Karin Dahl Assing, Richard W. Costello, Peter G. Gibson, Enrico Heffler, Jorge Máspero, Stefania Nicola, Diahn-Warng Perng (Steve), Francesca Puggioni, Sundeep Salvi, Chau-Chyun Sheu, Concetta Sirena, Camille Taillé, Tze Lee Tan, Leif Bjermer, Giorgio Walter Canonica, Takashi Iwanaga, Libardo Jiménez-Maldonado, Christian Taube, Luisa Brussino, David B. Price

**Affiliations:** ^1^ Department of Respiratory Medicine and Infectious Diseases, Research Unit, Bispebjerg Hospital, Copenhagen, Denmark; ^2^ Observational and Pragmatic Research Institute, Singapore, Singapore; ^3^ Optimum Patient Care Global, Cambridge, United Kingdom; ^4^ Department of Medicine, Centre for Lung Health, Vancouver General Hospital, Vancouver, BC, Canada; ^5^ Department of Medicine, The University of British Columbia, Vancouver, BC, Canada; ^6^ Faculty of Public Health, Medical University, Sofia, Bulgaria; ^7^ Woolcock Institute of Medical Research, The University of Sydney, Sydney, NSW, Australia; ^8^ School of Medicine, Sydney Campus, The University of Notre Dame, Sydney, NSW, Australia; ^9^ Centro de Excelencia en Asma y Alergia, Hospital Médica Sur, Ciudad de México, Mexico; ^10^ BioPharmaceuticals Medical, AstraZeneca, Gaithersburg, MD, United States; ^11^ Department of Pulmonology, King Fahad Medical City, Riyadh, Saudi Arabia; ^12^ College of Medicine, Alfaisal University, Riyadh, Saudi Arabia; ^13^ Macquarie Medical School, Faculty of Medicine, Health and Human Sciences, Macquarie University, Sydney, NSW, Australia; ^14^ Centre for Public Health, School of Medicine, Dentistry and Biomedical Sciences, Queen’s University Belfast, Belfast, United Kingdom; ^15^ Allergy, Asthma and Clinical Immunology Service, Alfred Health, Melbourne, VIC, Australia; ^16^ Public Health and Preventive Medicine, Monash University, Melbourne, VIC, Australia; ^17^ Respiratory Medicine Department, University of Ioannina, Ioannina, Greece; ^18^ Division of Infection, Immunity and Respiratory Medicine, University of Manchester, Manchester, United Kingdom; ^19^ Allergy Department, 2nd Pediatric Clinic, University of Athens, Athens, Greece; ^20^ Department of Respiratory Medicine, Barts Health National Health Services (NHS) Trust, London, United Kingdom; ^21^ Barts and The London School of Medicine and Dentistry, Queen Mary University of London, London, United Kingdom; ^22^ University Hospital St. Ivan Rilski, Sofia, Bulgaria; ^23^ Division of Pulmonary and Critical Care Medicine, Department of Internal Medicine, Seoul St. Mary’s Hospital, College of Medicine, The Catholic University of Korea, Seoul, Republic of Korea; ^24^ Respiratory Evaluation Sciences Program, Faculty of Pharmaceutical Sciences, The University of British Columbia, Vancouver, BC, Canada; ^25^ Division of Pulmonary and Critical Care Medicine, Department of Internal Medicine, Kaohsiung Medical University Hospital, Kaohsiung Medical University, Kaohsiung, Taiwan; ^26^ Department of Internal Medicine, School of Medicine, College of Medicine, Kaohsiung Medical University, Kaohsiung, Taiwan; ^27^ Department of Respiratory Medicine, Copenhagen ;University Hospital - Hvidovre, Copenhagen, Denmark; ^28^ Microbiology Department, College of Medicine, Kuwait University, Kuwait City, Kuwait; ^29^ Al-Rashed Allergy Center, Ministry of Health, Kuwait City, Kuwait; ^30^ Department of Pulmonology, University of Tartu and Lung Clinic, Tartu University Hospital, Tartu, Estonia; ^31^ Son Espases University Hospital-Institut d’Investigació Sanitària Illes Balears (IdISBa)-Ciberes, Mallorca, Spain; ^32^ Respiratory Research Unit, Bispebjerg University Hospital, Copenhagen, Denmark; ^33^ Center for Clinical Research and Prevention, Bispebjerg and Frederiksberg Hospital, Copenhagen, Denmark; ^34^ Wellcome-Wolfson Institute for Experimental Medicine, Queen’s University Belfast, Belfast, United Kingdom; ^35^ Respiratory Medicine at the School of Medicine, University of Nottingham, Nottingham, United Kingdom; ^36^ Division of Internal Medicine Asthma and Allergy, Medical University of Lodz, Lodz, Poland; ^37^ Division of Respiratory Medicine, Department of Internal Medicine, Kobe University Graduate School of Medicine, Kobe, Japan; ^38^ Department of Clinical and Experimental Sciences – University of Brescia, Spedali Civili di Brescia, Brescia, Italy; ^39^ Instituto Neumológico del Oriente, Bucaramanga, Colombia; ^40^ Centre Hospitalier Universitaire (CHU) Sart-Tilman, GIGA I3, University of Liege, Liège, Belgium; ^41^ Department of Medicine, National Jewish Health (NJH) Cohen Family Asthma Institute, National Jewish Health, Denver, CO, United States; ^42^ Department of Women’s and Children’s Health, Uppsala University, Uppsala, Sweden; ^43^ CINTESIS@RISE, MEDCIDS, Faculty of Medicine of the University of Porto, Porto, Portugal; ^44^ PhyMedExp, Univ Montpellier, National Center for Scientific Research (CNRS), The National Institute of Health and Medical Research (INSERM), Centre Hospitalier Universitaire (CHU) Montpellier, Montpellier, France; ^45^ Department of Respiratory Medicine, Ghent University Hospital, Ghent, Belgium; ^46^ Departments of Epidemiology and Respiratory Medicine, Erasmus Medical Center Rotterdam, Rotterdam, Netherlands; ^47^ Saw Swee Hock School of Public Health, National University of Singapore, Singapore, Singapore; ^48^ Department of Respiratory Medicine, Fiona Stanley Hospital, Perth, WA, Australia; ^49^ Department of Medicine, Central Clinical School, Monash University, Melbourne, VIC, Australia; ^50^ Division of Allergy and Clinical Immunology, Department of Medicine, National Jewish Health, Denver, CO, United States; ^51^ Guy’s Severe Asthma Centre, Guy’s Hospital, King’s College London, London, United Kingdom; ^52^ Department of Medicine, Lung Institute, Makerere University Lung Institute, Kampala, Uganda; ^53^ Department of Respiratory and Critical Care Medicine, Singapore General Hospital, Singapore, Singapore; ^54^ Medical University of Lodz, Lodz, Poland; ^55^ Allergy Centre, Tampere University Hospital, Tampere, Finland; ^56^ Faculty of Medicine and Health Technology, Tampere University, Tampere, Finland; ^57^ FAST and Chronic Programmes, Alexandra Hospital, National University Health System, Singapore, Singapore; ^58^ Division of Respiratory and Critical Care Medicine, Department of Medicine, National University Hospital, National University Health System, Singapore, Singapore; ^59^ Rashid Hospital, Dubai Health Authority (DHA), Dubai, United Arab Emirates; ^60^ Dubai Academic and Health Corporation, Dubai, United Arab Emirates; ^61^ Department of Respiratory Medicine, University of Leicester, Leicester, United Kingdom; ^62^ BioPharmaceutical Medical, AstraZeneca, Cambridge, United Kingdom; ^63^ Lung Division, Royal Brompton and Harefield Hospital, London, United Kingdom; ^64^ Department of Respiratory and Critical Care Medicine, Tan Tock Seng Hospital, Singapore, Singapore; ^65^ 2nd Respiratory Medicine Department, National and Kapodistrian University of Athens Medical School, Attikon University Hospital, Athens, Greece; ^66^ Respiratory Medicine, Royal Brompton Hospital, London, United Kingdom; ^67^ Pneumology Service, Lucus Augusti University Hospital, Sergas (Galician Healthcare Service) Integrated Management Structure (EOXI) Lugo, Cervo, Spain; ^68^ Department of Thoracic Medicine, Concord Hospital, Sydney, NSW, Australia; ^69^ Faculty of Medicine, Health and Human Sciences, Macquarie University, Sydney, NSW, Australia; ^70^ Allergy and Clinical Immunology, G. Martino Hospital, University of Messina, Messina, Italy; ^71^ Pulmonary Unit, Hospital Universitario San Ignacio, Bogotá, Colombia; ^72^ School of Medicine, Pontificia Universidad Javeriana, Bogotá, Colombia; ^73^ Department of Respiratory and Critical Care Medicine, Changi General Hospital, Singapore, Singapore; ^74^ Centro Internacional de Investigación en Neumología (CINEUMO), Respiratory Research Center, Fundación Neumológica Colombiana, Bogotá, Colombia; ^75^ Universidad de La Sabana, Doctoral Biosciences, Chia, Colombia; ^76^ Division of Allergy and Clinical Immunology, Department of Medicine, University of Colorado School of Medicine, Aurora, CO, United States; ^77^ Department of Medicine and Surgery, University of Insubria, Varese, Italy; ^78^ Health Services and Outcomes Research, National Healthcare Group, Singapore, Singapore; ^79^ Lee Kong Chian School of Medicine, Nanyang Technological University, Singapore, Singapore; ^80^ Department of Respiratory Medicine, Aalborg University Hospital, Aalborg, Denmark; ^81^ Department of Respiratory Medicine, Clinical Research Centre, Smurfit Building Beaumont Hospital, Royal College of Surgeons Ireland (RCSI), Dublin, Ireland; ^82^ Australian Severe Asthma Network, Priority Research Centre for Healthy Lungs, University of Newcastle, Newcastle, NSW, Australia; ^83^ Department of Respiratory and Sleep Medicine, Hunter Medical Research Institute, John Hunter Hospital, Newcastle, NSW, Australia; ^84^ Personalized Medicine, Asthma and Allergy, Istituto Clinico Humanitas, Humanitas Cancer Center (IRCCS) Humanitas Research Hospital, Rozzano, Italy; ^85^ Department of Biomedical Sciences, Humanitas University, Pieve Emanuele, Italy; ^86^ Clinical Research for Allergy and Respiratory Medicine, CIDEA Foundation, Buenos Aires, Argentina; ^87^ University Career of Specialists in Allergy and Clinical Immunology at the Buenos Aires University School of Medicine, Buenos Aires, Argentina; ^88^ Allergy and Immunology Unit, L'Azienda Ospedaliera (AO) Ordine Mauriziano di Torino, Turin, Italy; ^89^ School of Medicine, National Yang Ming Chiao Tung University, Taipei, Taiwan; ^90^ Department of Chest Medicine, Taipei Veterans General Hospital, Taipei, Taiwan; ^91^ Pulmocare Research and Education Foundation, Pune, India; ^92^ Severe Asthma Network Italy (SANI), Milano, Italy; ^93^ Department of Respiratory Diseases, Bichat Hospital, l'Assistance publique – Hôpitaux de Paris (AP-HP) Nord-Université Paris Cité, Paris, France; ^94^ Department of Family Medicine, National University Health System, Singapore, Singapore; ^95^ Respiratory Medicine and Allergology, Department of Clinical Sciences, Skåne University Hospital, Lund University, Lund, Sweden; ^96^ Kindai University Hospital, Osakasayama, Japan; ^97^ Fundación Neumológica Colombiana, ASMAIRE REXPIRA (Atención integral y rehabilitación en asma or Comprehensive Care and Rehabilitation in Asthma) Program, Bogotá, Colombia; ^98^ Department of Pulmonary Medicine, University Medical Center Essen-Ruhrlandklinik, Essen, Germany; ^99^ Department of Medical Sciences, University of Turin, Turin, Italy; ^100^ Centre of Academic Primary Care, Division of Applied Health Sciences, University of Aberdeen, Aberdeen, United Kingdom

**Keywords:** severe asthma, biomarkers, eosinophil (EOS), FeNO (Fraction of exhaled Nitric Oxide), biologics, FEV1, personalized medicine (PM)

## Abstract

**Background:**

To date, studies investigating the association between pre-biologic biomarker levels and post-biologic outcomes have been limited to single biomarkers and assessment of biologic efficacy from structured clinical trials.

**Aim:**

To elucidate the associations of pre-biologic individual biomarker levels or their combinations with pre-to-post biologic changes in asthma outcomes in real-life.

**Methods:**

This was a registry-based, cohort study using data from 23 countries, which shared data with the International Severe Asthma Registry (May 2017-February 2023). The investigated biomarkers (highest pre-biologic levels) were immunoglobulin E (IgE), blood eosinophil count (BEC) and fractional exhaled nitric oxide (FeNO). Pre- to approximately 12-month post-biologic change for each of three asthma outcome domains (i.e. exacerbation rate, symptom control and lung function), and the association of this change with pre-biologic biomarkers was investigated for individual and combined biomarkers.

**Results:**

Overall, 3751 patients initiated biologics and were included in the analysis. No association was found between pre-biologic BEC and pre-to-post biologic change in exacerbation rate for any biologic class. However, higher pre-biologic BEC and FeNO were both associated with greater post-biologic improvement in FEV_1_ for both anti-IgE and anti-IL5/5R, with a trend for anti-IL4Rα. Mean FEV_1_ improved by 27-178 mL post-anti-IgE as pre-biologic BEC increased (250 to 1000 cells/µL), and by 43-216 mL and 129-250 mL post-anti-IL5/5R and -anti-IL4Rα, respectively along the same BEC gradient. Corresponding improvements along a FeNO gradient (25-100 ppb) were 41-274 mL, 69-207 mL and 148-224 mL for anti-IgE, anti-IL5/5R, and anti-IL4Rα, respectively. Higher baseline BEC was also associated with lower probability of uncontrolled asthma (OR 0.392; p=0.001) post-biologic for anti-IL5/5R. Pre-biologic IgE was a poor predictor of subsequent pre-to-post-biologic change for all outcomes assessed for all biologics. The combination of BEC + FeNO marginally improved the prediction of post-biologic FEV_1_ increase (adjusted R^2^: 0.751), compared to BEC (adjusted R^2^: 0.747) or FeNO alone (adjusted R^2^: 0.743) (p=0.005 and <0.001, respectively); however, this prediction was not improved by the addition of IgE.

**Conclusions:**

The ability of higher baseline BEC, FeNO and their combination to predict biologic-associated lung function improvement may encourage earlier intervention in patients with impaired lung function or at risk of accelerated lung function decline.

## Introduction

Severe asthma is a heterogenous syndrome encompassing several clinical phenotypes and endotypes, or patterns of airway inflammation (1, 2). The type-2 (T2)-inflammatory endotype, associated with increased blood eosinophil count (BEC) and/or fractional exhaled nitric oxide (FeNO) concentrations or total immunoglobulin E (IgE) and specific IgE, is estimated to account for up to 80% of adults with severe asthma using an algorithm informed by these biomarkers (and clinical characteristics) and developed by expert consensus (2–4). Investigation of how these biomarkers may be associated with better asthma outcomes in patients who initiate biologics or other therapies, and their use in guiding asthma treatment-related decisions is an area of intense research (5–12). However, the effectiveness of T2-directed biologics is variable even in patients with similar biomarker profiles (13). The challenge, therefore, remains to further unravel asthma endotypes within the T2-high severe asthma population and to accurately select patients who will respond best to the selected biologic therapy; matching the right patient to the right biologic in the course of their disease and allowing for a more personalized and targeted approach to asthma treatment. Use of biomarkers to identify non-responders is arguably just as important to avoid unnecessary treatment.

However, there are still many issues with T2 biomarkers in terms of how best to measure and interpret them, as well as with the evidence underpinning their utility to assess and predict response to biologics in patients with severe asthma. For example, biomarker levels show marked temporal variability, are influenced by site of measurement, and must be interpreted in the context of background corticosteroid treatment and treatment adherence (14, 15). Biomarkers also frequently overlap, with their utility considered by some as a means to identify severe asthma rather than select biologic therapy (16). There are also gaps in our knowledge about their relationship with each other (2). Biomarker cut-off values to inform biologic eligibility have also been influenced by randomized clinical trial (RCT) criteria, rather than by studies specifically designed to investigate the utility of biomarker level to predict biologic response in real life, and show marked inter-country variability, indicative of variable interpretation of the same evidence by different regulators and reimbursement bodies (17). There is clearly a need for biomarker validation in terms of predicting response to therapy (18). A clinically applicable biomarker should be ‘Superior’ (outperform current practice), ‘Actionable’ (change patient management), ‘Valuable’ (improve patient outcomes), ‘Economical’ (cost-saving or cost-effective) and ‘clinically Deployable’ (analysis technology available in clinical laboratory) (i.e., the SAVED approach) (18).

To date, previous clinical studies that examined the influence of pre-biologic biomarker levels on post-biologic outcomes have compared biologic effectiveness, stratified by biomarker concentration compared to a placebo group, rather than within a biologic-treated group (i.e. compared to baseline). Whether this relationship is seen when pre-to-post biologic effect is assessed along a pre-biologic biomarker concentration gradient remains to be determined; proof of such a relationship would be more meaningful to clinicians when deciding which biologic to prescribe for which patient. Furthermore, the ability of biomarkers to predict which patients will experience improved lung function is not well-studied (19–21). This is arguably a more valuable endpoint considering that many patients with asthma experience significant irreversible deterioration of their lung function over time, (which is associated with severe disease), that lung function declines more quickly in younger adults compared to older patients who have had the same number of exacerbations (22–24), and that those with better lung function are more likely to achieve asthma remission when treated with biologics (25).

Previous research conducted by the International Severe Asthma Registry (ISAR; https://isaregistries.org/) has shown considerable overlap of inflammatory biomarkers in severe asthma, suggesting that a more comprehensive approach may be needed to identify the best therapy for patients, rather than reliance on a single biomarker threshold positivity (2). ISAR is the largest, real-world data repository of severe asthma cases, including data on >17,000 patients from 25 countries. It offers a unique opportunity to investigate the relationship between biomarker profile and pre-to- post biologic change across a range of biomarkers, asthma outcomes, and biologic classes in a real-world setting that includes patients who would not qualify for entry into RCTs (26–28). Our aim was to investigate T2 inflammatory biomarker distribution and correlations, the association of pre-biologic biomarker levels with pre-to-post biologic change in asthma outcomes, and whether combined biomarker measurements lead to an improved association with pre-to-post biologic change.

## Methods

### Study design and data source

This was a registry-based cohort study using data from ISAR (https://isaregistries.org/), the largest adult severe asthma registry in the world (26). Patients with severe asthma included in ISAR have been well characterized (29) and phenotyped (3). The details of this registry have been described elsewhere (27), and details are provided in the Online Supplement. In this study we have included data from 23 countries (Argentina, Australia, Bulgaria, Canada, Colombia, Denmark, Greece, India, Ireland, Italy, Japan, Korea, Kuwait, Mexico, Poland, Portugal, Saudi Arabia, Singapore, Spain, Taiwan, United Arab Emirates, United Kingdom, and United States) that shared data with ISAR between May 1, 2017 and February 24, 2023. The study had two parts. The first part investigated pre-biologic biomarker level distribution and correlations for all patients, regardless of whether a biologic was subsequently initiated. The second part investigated whether an association exists between pre-biologic biomarker levels and change in asthma outcomes pre-to-post biologic in those patients who initiated biologics. For this part, study entry corresponded to date of first biologic initiation, and asthma-related outcomes were assessed both in the 1-year period pre- and post-biologic therapy (**Supplementary Figure 1**; **Table 1**).

**Table 1:** Asthma outcome definitions and Asthma outcome definitions and timing of outcome assessments pre- and post-biologic.

OUTCOME	Pre-biologic	Post-biologic
Exacerbation rate (count per year)	Number of exacerbations requiring rescue steroids in the 12 months preceding biologic initiation	Number of exacerbations per year requiring rescue steroids after biologic initiation during the available 12-month follow-up period (min. 48 weeks)
post-bronchodilator FEV_1_	Highest reading in the 12 months preceding biologic initiation	Assessed closest to 1 year after biologic initiation (min. 24 weeks)
Asthma control^a^	Well-, partly, uncontrolled in the 12 months preceding biologic initiation	

a Control categories defined by Global Initiative for Asthma 2020 update (30). For countries providing Asthma Control Questionnaire (ACQ) or Asthma Control Test (ACT) scores to rate asthma control instead of Global Initiative for Asthma (GINA) control categories, conversions were performed as follows: mean ACQ score ≤0.75 =well-controlled, mean ACQ score >0.75 to <1.5 = partly controlled, mean ACQ score ≥1.5 = uncontrolled; total ACT score >19 = well-controlled, total ACT score >15 to ≤19 = partly controlled, total ACT score ≤15 = uncontrolled.

FEV_1_, forced expiratory volume in one second.

### Patients

All patients were enrolled in ISAR and were required to be aged ≥18 years and have severe asthma (consistently defined as receiving treatment at Global Initiative for Asthma [GINA] 2018 Step 5 or with uncontrolled asthma at GINA Step 4) (31). Subsequent inclusion criteria differed by analysis. For the biomarker distribution and correlation analyses, patients were also required to have a pre-biologic value for any of the biomarkers assessed (i.e., IgE, BEC, or FeNO). For the association of individual biomarkers and pre-to-post biologic change analyses, patients also must have received a biologic and have pertinent information on at least one asthma outcome (i.e., exacerbations, lung function, or asthma control). To be able to attribute any associations with a particular biologic type, patients who switched biologic therapies during follow-up were excluded from these analyses. Finally, for inclusion in the association of multiple biomarkers and pre-to-post biologic change analyses, patients were also required to have pre-biologic values for all three investigated biomarkers. Those who had bronchial thermoplasty were excluded.

### Variables

Collected variables included pre-biologic demographic and clinical characteristics (**Table 2**); highest pre-biologic values for FeNO (ppb), IgE (IU/mL), and BEC (cells/µL); and asthma outcomes pre- and post-biologic therapy. For the distribution and correlation analyses, we used the highest pre-biologic biomarker value in the 1-year period prior to biologic initiation (for those who subsequently initiated a biologic) or the highest value at any time for those who did not subsequently initiate a biologic (because all values were pre-biologic). For the association analyses, the highest pre-biologic biomarker value in the 1-year period prior to biologic initiation was used. Median biomarker concentrations in the first 3 months biologic therapy and subsequently at >3-12, >12-24, >24-36 and >36 months post-biologic initiation were also collected to investigate biomarker temporal stability.

**Table 2:** Pre-biologic characteristics of patients included in association of T2-biomarkers and pre-to post biologic outcome analyses.

	Total (N=3751)	Biologic class		
		Anti-IL5/5R (N=1,983)	Anti-IgE (N=1,340)	Anti-IL4Rα (N=428)
Sex	N=3743	N=1982	N=1338	N=428
Female, n (%)	2,345 (62.6%)	1,192 (60.1)	888 (66.4)	265 (61.9)
Age at index date, yrs				
Mean (SD)	52.9 (14.5)	55.2 (13.9)	50.1 (14.5)	50.7 (15.2)
Ethnicity	N=3275	N=1730	N=1180	N=365
White, n (%)	2,662 (81.3)	1,444 (83.5)	943 (79.9)	275 (75.3)
South-East Asian, n (%)	91 (2.8)	48 (2.8)	27 (2.3)	16 (4.4)
North-East Asian, n (%)	107 (3.3)	62 (3.6)	25 (2.1)	20 (5.5)
African, n (%)	82 (2.5)	44 (2.5)	29 (2.5)	9 (2.5)
Mixed, n (%)	85 (2.6)	15 (0.9)	57 (4.8)	13 (3.6)
Other, n (%)	248 (7.6)	117 (6.8)	99 (8.4)	32 (8.8)
Smoking status, n (%)	N=1641	N=1002	N=552	N=87
Current, n (%)	41 (2.5)	22 (2.2)	18 (3.3)	1 (1.1)
Ex-smoker, n (%)	451 (27.5)	315 (31.4)	106 (19.2)	30 (34.5)
Never smoked, n (%)	1149 (70.0)	665 (66.4)	428 (77.5)	56 (64.4)
Age at asthma onset, years	N=2245	N=1318	N=802	N=125
Mean (SD)	29.8 (18.3)	32.5 (18.2)	25.6 (17.4)	27.7 (19.4)
Age group at asthma onset, years	N=2,245	N=1,318	N=802	N=125
<18, n (%)	661 (29.4)	313 (23.7)	303 (37.8)	45 (36.0)
18-40, n (%)	916 (40.8)	541 (41.0)	334 (41.6)	41 (32.8)
41-64, n (%)	609 (27.1)	419 (31.8)	154 (19.2)	36 (28.8)
65+, n (%)	59 (2.6)	45 (3.4)	11 (1.4)	3 (2.4)
Duration of asthma, years	N=2,245	N=1,318	N=802	N=125
Median (IQR)	19.8 (10.0-34.6)	19.0 (9.0-33.7)	20.4 (11.0-35.8)	20.5 (9.0-35.0)
Baseline BEC, cells/µL	N=3,195	N=1,727	N=1,080	N=388
Median (IQR)	400 (200-640)	475 (260-730)	245 (100-500)	400 (200-600)
Baseline FeNO, ppb	N=1,885	N=1,082	N=537	N=267
Median (IQR)	32.0 (17.0-60.0)	38.0 (20.0-68.0)	23.0 (13.0-44.0)	33.0 (17.0-64.0)
Baseline IgE, IU/mL	N=2,754	N=1,378	N=1,072	N=304
Median (IQR)	172 (68-410)	127 (50-319)	262 (118-528)	123 (37-320)
One or more allergies detected	N=2,174	N=1,033	N=922	N=219
Yes, n (%)	1460 (67.2)	571 (55.3)	753 (81.7)	136 (62.1)
Baseline asthma control^a^	N=1,780	N=1,096	N=586	N=98
Well controlled, n (%)	227 (12.8)	128 (11.7)	83 (14.2)	16 (16.3)
Partly controlled, n (%)	342 (19.2)	223 (20.3)	93 (15.9)	26 (26.5)
Uncontrolled, n (%)	1211 (68.0)	745 (68.0)	410 (70.0)	56 (57.1)
Baseline FEV_1_, L	N=2,840	N=1,522	N=990	N=328
Mean (SD)	2.1 (0.8)	2.1 (0.8)	2.1 (0.8)	2.3 (0.9)
Baseline ppFEV_1`_	N=2,564	N=1,392	N=912	N=260
Mean (SD)	73.0 (24.7)	72.3 (24.6)	72.8 (24.9)	77.7 (24.2)
Annualized baseline exacerbation rate	N=3,080	N=1,633	N=1,075	N=372
Mean (SD)	2.2 (2.9)	2.7 (3.2)	1.8 (2.6)	0.8 (1.6)
Eosinophilic grade^b^	N=3,451	N=1,983	N=1,080	N=388
Unlikely, n (%)	179 (5.2)	0 (0.0)	145 (13.4)	34 (8.8)
Least likely, n (%)	250 (7.2)	0 (0.0)	198 (18.3)	52 (13.4)
Likely, n (%)	201 (5.8)	0 (0.0)	160 (14.8)	41 (10.6)
Most likely, n (%)	2,821 (81.7)	1,983 (100.0)	577 (53.4)	261 (67.3)
Comorbidities				
	N=3,684	N=1,957	N=1,303	N=424
Atopic dermatitis, n (%)	482 (13.1)	203 (10.4)	190 (14.6)	89 (21.0)
	N=2,466	N=1,260	N=967	N=239
Allergic rhinitis, n (%)	1743 (70.7)	773 (61.3)	765 (79.1)	205 (85.8)
	N=2,382	N=1,371	N=815	N=196
CRSwNP, n (%)	1073 (45.0)	697 (50.8)	236 (29.0)	140 (71.4)
CRS, n (%)	1470 (61.7)	928 (67.7)	384 (47.1)	158 (80.6)
Receiving LTOCS at Bx initiation	N=3,704	N=1,953	N=1,324	N=427
Yes, n (%)	1006 (27.2)	675 (34.6)	264 (19.9)	67 (15.7)
Mean Daily LTOCS dose, mg Mean (SD) Median (IQR)	N=867 12.8 (24.5) 10 (5, 15)	N=574 12.8 (27.2) 10 (5, 15)	N=229 13.4 (20.2) 10 (5, 15)	N=64 10.5 (7.4) 10 (5, 15)
Add-on therapies to ICS/LABA	N=3,731	N=1,983	N=1,320	N=428
LAMA, n (%)	1,038 (27.8)	540 (27.2)	324 (24.5)	174 (40.7)
LTRA, n (%)	1,286 (34.5)	543 (27.4)	522 (39.5)	221 (51.6)
Macrolides, n (%)	183 (4.9)	71 (3.6)	67 (5.1)	45 (10.5)
Theophylline, n (%)	179 (4.8)	102 (5.1)	67 (5.1)	10 (2.3)

a GINA 2020 control categories (30). For countries providing Asthma Control Questionnaire (ACQ) or Asthma Control Test (ACT) scores to rate asthma control instead of GINA control categories, conversions were performed as follows: mean ACQ score ≤0.75 =well-controlled, mean ACQ score >0.75 to <1.5 = partly controlled, mean ACQ score ≥1.5 = uncontrolled; total ACT score >19 = well-controlled, total ACT score >15 to ≤19 = partly controlled, total ACT score ≤15 = uncontrolled.

b Categorized according to the eosinophil phenotype gradient algorithm (3).

BEC, blood eosinophil count; Bx, biologic; CRS, chronic rhinosinusitis; CRSwNP, chronic rhinosinusitis with nasal polyps; FeNO, fractional exhaled nitric oxide; FEV_1_, post-bronchodilator forced expiratory volume in one second; GINA, Global Initiative for Asthma; ICS, inhaled corticosteroid; IgE, immunoglobulin E; IL, interleukin; IQR, interquartile range; LABA, long-acting β2-agonist; LAMA, long-acting muscarinic antagonist; LTRA, leukotriene antagonists; LTOCS, long-term oral corticosteroid; ppb, part per billion; ppFEV_1_, percent predicted forced expiratory volume in one second; SD, standard deviation.

Asthma outcomes investigated included annual exacerbation rate, highest post-bronchodilator forced expiratory volume in one second (FEV_1_), and asthma control (**Table 1**). Asthma control was categorized as well-, partly, or uncontrolled according to GINA 2020 criteria (30). If contributing countries used the Asthma Control Test (ACT) (32) or Asthma Control Questionnaire (ACQ) (33) to assess asthma control, conversions were made to fit the GINA control categories as follows: mean ACQ score: well controlled (≤0.75); partly controlled (>0.75 to <1.5), uncontrolled (≥1.5); and total ACT score: well controlled (>19); partly controlled (>15 to ≤19), uncontrolled (≤15). Similar cut-offs and correlations have been described (34, 35) and used by others (36–38).

### Study outcomes and statistics

Correlations of pre-biologic biomarker values collected within 7 days of each other (for patients who had pairs of biomarkers collected at similar times: BEC/FeNO, BEC/IgE and FeNO/IgE) were tested using Pearson’s correlation using log_10_ values of the biomarkers. Changes in biomarkers in the first 3 months and at >3-12, >12-24, >24-36 and >36 months post-biologic initiation were summarized descriptively.

The association between individual pre-biologic T2 biomarkers and pre-to-post biologic change (by biologic class) was assessed for each of the 3 asthma outcomes using regression models with follow-up outcomes as the response, adjusting for baseline level of the relevant outcome. Linear, negative binomial, and logistic regression models were used for FEV_1_, exacerbations, and asthma control, respectively, according to the type and distribution of the data. Follow-up rates, means, or probabilities were estimated using adjusted predictions from the models for selected values of the biomarkers. For exacerbations the estimated follow-up rates were calculated for patients with a baseline exacerbation rate of 2.2 per year (mean value in the biologic population), and for FEV_1_ the estimated follow-up values were calculated for patients with a baseline FEV_1_ of 2.1 L (mean value in the biologic population). These estimates were plotted in the figures as the change from baseline by subtracting the baseline value at which they were evaluated (2.2 exacerbations per year, or FEV_1_ = 2.1 L). For asthma control, the adjusted predictions for the probability of uncontrolled asthma at follow-up were estimated for each biologic type assuming the proportion of patients with uncontrolled asthma at baseline was 68% (equal to the proportion amongst all included patients). This was equivalent to estimating the rate of uncontrolled asthma at follow-up in each biologic class as if all included patients had received that biologic. Separate models were fitted for BEC, FeNO and IgE. Analyses for each post-biologic asthma outcome (for all biologic classes combined) were also stratified by various subgroups: long-term oral corticosteroid (LTOCS) at baseline (yes/no), age of asthma onset (<18/≥18 years), presence of allergy (yes/no), and baseline annual exacerbation burden (0/≥1 exacerbation).

The association of multiple biomarkers and pre-to-post biologic change in asthma outcomes was assessed using the same statistical models as described above for each asthma outcome, but also including the additional biomarker(s) as independent variables. The accuracy of the predictions made by the model was assessed using: (i) percentage of variance in follow-up FEV_1_ explained by the model (adjusted R^2^); (ii) percentage of uncontrolled asthma outcomes correctly classified by the model (probability > 0.5 = positive outcome predicted, probability < 0.5 = negative outcome predicted); and (iii) mean absolute error in the exacerbation rate predicted by the model compared with the actual follow-up rate. The increase in model fit due to the incorporation of additional biomarkers was tested using likelihood ratio tests. For the regression analyses, positive outlying baseline biomarkers were excluded (defined as > upper quartile + 1.5 x inter-quartile range) (39) to avoid fitting the regressions for a range of biomarker values where there was insufficient coverage in the data. All analyses were undertaken in STATA v15.1, and p-values ≤0.05 were considered statistically significant. We also plotted point estimates from the regression models for the lung function outcome along a BEC plus FeNO gradient and tabulated mean values for pre-to-post biologic change in each asthma outcome assessed according to FeNO/BEC combination categories ranging from low/low (25 ppb/<150 cells/µL) to high/high (>50 ppb/>300 cells/µL).

## Results

### Subject disposition

As of February 24, 2023, ISAR included data on 15,154 adult patients with severe asthma. A total of 11,363 patients from 23 countries were eligible for assessment of biomarker distribution and correlation. Of these, 3,751 were eligible for inclusion in the association of biomarker with biologic effectiveness analyses, of whom 1,983 initiated anti–interleukin-5/5 receptor (anti-IL5/5R) therapy, 1,340 initiated anti-IgE therapy, and 428 initiated anti–interleukin-4 receptor alpha (anti-IL4Rα) therapy (**Supplementary Figure 2**). A total of 1,323 patients had data available for all three biomarkers (BEC, FeNO and IgE) and were included in the association of multiple biomarkers with biologic effectiveness analyses.

### Pre-biologic demographic and clinical characteristics

**Table 2** summarizes baseline characteristics for those patients included in the analyses investigating the association between pre-biologic biomarker concentration and pre-to-post biologic changes in outcomes (n=3,751). These patients tended to be middle-aged (mean [standard deviation; SD] 52.9 [14.5] years), were predominantly female (62.6%) and White (81.3%), and had adult-onset disease (70.6%). Pre-biologic biomarker concentrations (BEC, FeNO, and IgE) were all elevated, and 81.7% had an eosinophilic asthma phenotype. Morbidity burden was high, evidenced by a mean (SD) exacerbation rate/year of 2.2 (2.9) and percent predicted FEV_1_ (ppFEV_1_) of 73.0% pre-biologic initiation, and a high proportion of patients with uncontrolled disease (68.0%) and receiving LTOCS (27.2%). Overall, the mean (SD) daily dose of LTOCS (prednisone equivalent dosing) was 12.8 (24.5) mg pre-biologic and 8.7 (24.3) mg post-biologic (anti-IgE therapy: 8.9 [18.9] mg; anti-IL5/5R therapy: 8.9 [27.2] mg; anti-IL4Rα therapy: 6.2 [7.4] mg), which was a mean (SD) daily decrease of 4.1 (8.2) mg. Potentially T2-related comorbidities were prevalent, notably allergic rhinitis (70.7%), atopic dermatitis (13.1%), and chronic rhinosinusitis with nasal polyps (45.0%). Compared to patients treated with anti-IgE therapy, those who subsequently received anti-IL5/5R therapy tended to have later onset disease and have more severe asthma in terms of exacerbation rate and LTOCS use. Patients treated with anti-IL4Rα therapy tended to have the least severe disease; however, all subsequent analyses were adjusted for baseline for all asthma outcomes assessed. Pre-biologic BEC and FeNO concentrations were elevated in the anti-IL5/5R and anti-IL4Rα biologic groups, whereas patients who subsequently initiated anti-IgE therapy tended to have higher IgE concentrations and one or more allergies. Pre-biologic characteristics (overall and by biologic class) for patients included in the assessment of biologic distribution and correlation analyses are provided in **Supplementary Table 1**.

### Biomarker distributions, temporal stability and correlations

Median (interquartile range) values for BEC, FeNO and IgE were 300 (200-600) cells/µL, 29.0 (15.0-60.0) ppb and 158 (52-457) IU/mL, respectively (**Supplementary Figure 3**) and were generally higher in patients who subsequently initiated a biologic (**Supplementary Table 1**). BEC and FeNO decreased following treatment with anti-IL5/5R and anti-IL4Rα therapies, respectively. Anti-IgE therapy had little effect on these biomarkers (**Supplementary Figure 4**; **Supplementary Tables 2**, **3**). Positive correlations between all pairs of biomarkers measured within 7 days of each other were noted, although the strength of the correlations was low (r ≤0.4) in all cases (**Supplementary Figures 5A-C**), but stronger in those not receiving LTOCS (**Supplementary Table 4**).

### Associations

**Figure 1:**
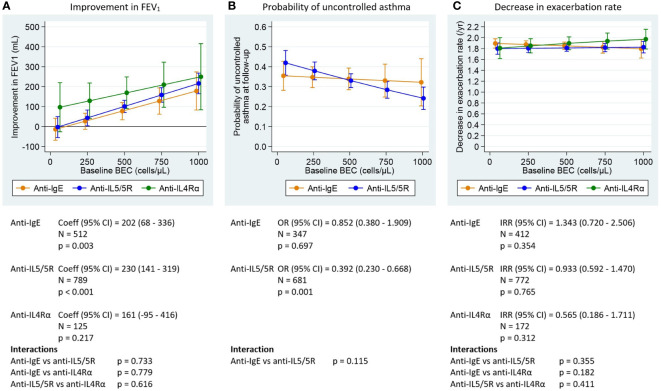
Association between pre-biologic BEC and post-biologic asthma outcomes, by biologic class adjusted for baseline for each outcome. BEC, blood eosinophil; FEV_1_, post-bronchodilator forced expiratory volume in one second; IgE, immunoglobulin E Graphs show point estimates from the regression models for selected values of the biomarkers. **(A)** change in FEV_1_ for a patient with baseline FEV_1_ = 2.1 L (mean baseline FEV_1_ for the biologic patients in ISAR), **(B)** asthma control at follow-up for a population with 68% uncontrolled asthma at baseline (proportion for biologic patients in ISAR), **(C)** change in exacerbation rate for a patient with 2.2 exacerbations per year at baseline (mean baseline exacerbation rate for the biologic patients in ISAR). Asthma control assessed using Global Initiative for Asthma (GINA) 2020 control categories (30). For countries providing Asthma Control Questionnaire (ACQ) or Asthma Control Test (ACT) scores to rate asthma control instead of GINA control categories, conversions were performed as follows: mean ACQ score ≤0.75 =well-controlled, mean ACQ score >0.75 to <1.5 = partly controlled, mean ACQ score ≥1.5 = uncontrolled; total ACT score >19 = well-controlled, total ACT score >15 to ≤19 = partly controlled, total ACT score ≤15 = uncontrolled. Coefficients, odds ratios, and incidence rate ratios are the estimated change in the outcome per 1000 cells/µL (BEC), per 100 ppb (FeNO) or per 1000 IU/mL (IgE). P-values are for tests of association between the outcomes after treatment and baseline levels of the biomarkers (adjusted for baseline level of the outcome). Interaction tests are for comparisons of the slope coefficients for the different biologic classes, estimated by the model.

**Figure 2:**
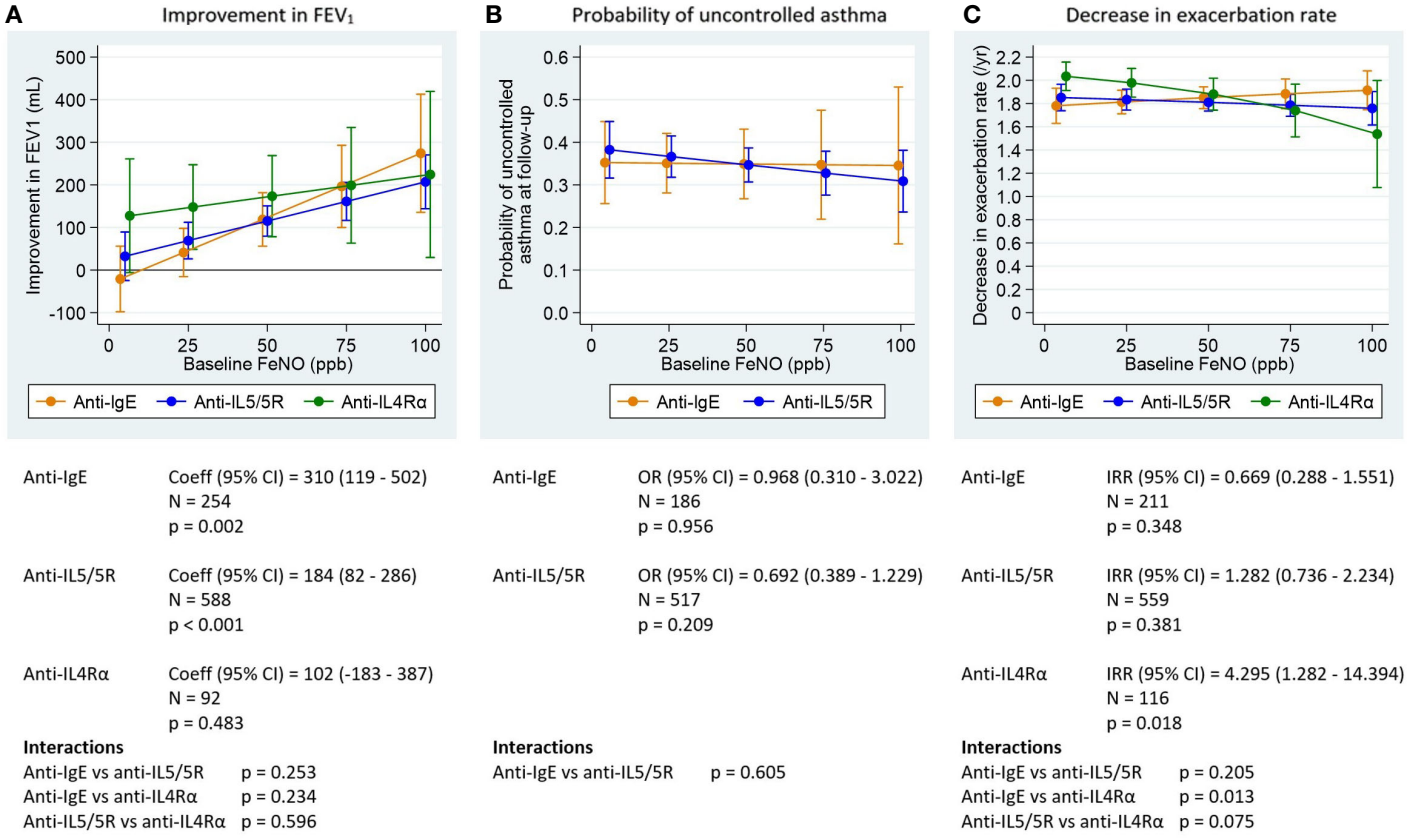
Association between pre-biologic FeNO and post-biologic asthma outcomes by biologic class, adjusted for baseline for each outcome. FeNO, fractional exhaled nitric oxide; FEV_1_, post-bronchodilator forced expiratory volume in one second; IgE, immunoglobulin E; ppb, parts per billion. Graphs show point estimates from the regression models for selected values of the biomarkers. **(A)** change in FEV_1_ for a patient with baseline FEV_1_ = 2.1 L (mean baseline FEV_1_ for the biologic patients in ISAR), **(B)** asthma control at follow-up for a population with 68% uncontrolled asthma at baseline (proportion for biologic patients in ISAR), **(C)** change in exacerbation rate for a patient with 2.2 exacerbations per year at baseline (mean baseline exacerbation rate for the biologic patients in ISAR). Asthma control assessed using Global Initiative for Asthma (GINA) 2020 control categories (30). For countries providing Asthma Control Questionnaire (ACQ) or Asthma Control Test (ACT) scores to rate asthma control instead of GINA control categories, conversions were performed as follows: mean ACQ score ≤0.75 =well-controlled, mean ACQ score >0.75 to <1.5 = partly controlled, mean ACQ score ≥1.5 = uncontrolled; total ACT score >19 = well-controlled, total ACT score >15 to ≤19 = partly controlled, total ACT score ≤15 = uncontrolled. Coefficients, odds ratios, and incidence rate ratios are the estimated change in the outcome per 1000 cells/µL (BEC), per 100 ppb (FeNO) or per 1000 IU/mL (IgE). P-values are for tests of association between the outcomes after treatment and baseline levels of the biomarkers (adjusted for baseline level of the outcome). Interaction tests are for comparisons of the slope coefficients for the different biologic classes, estimated by the model.

**Figure 3:**
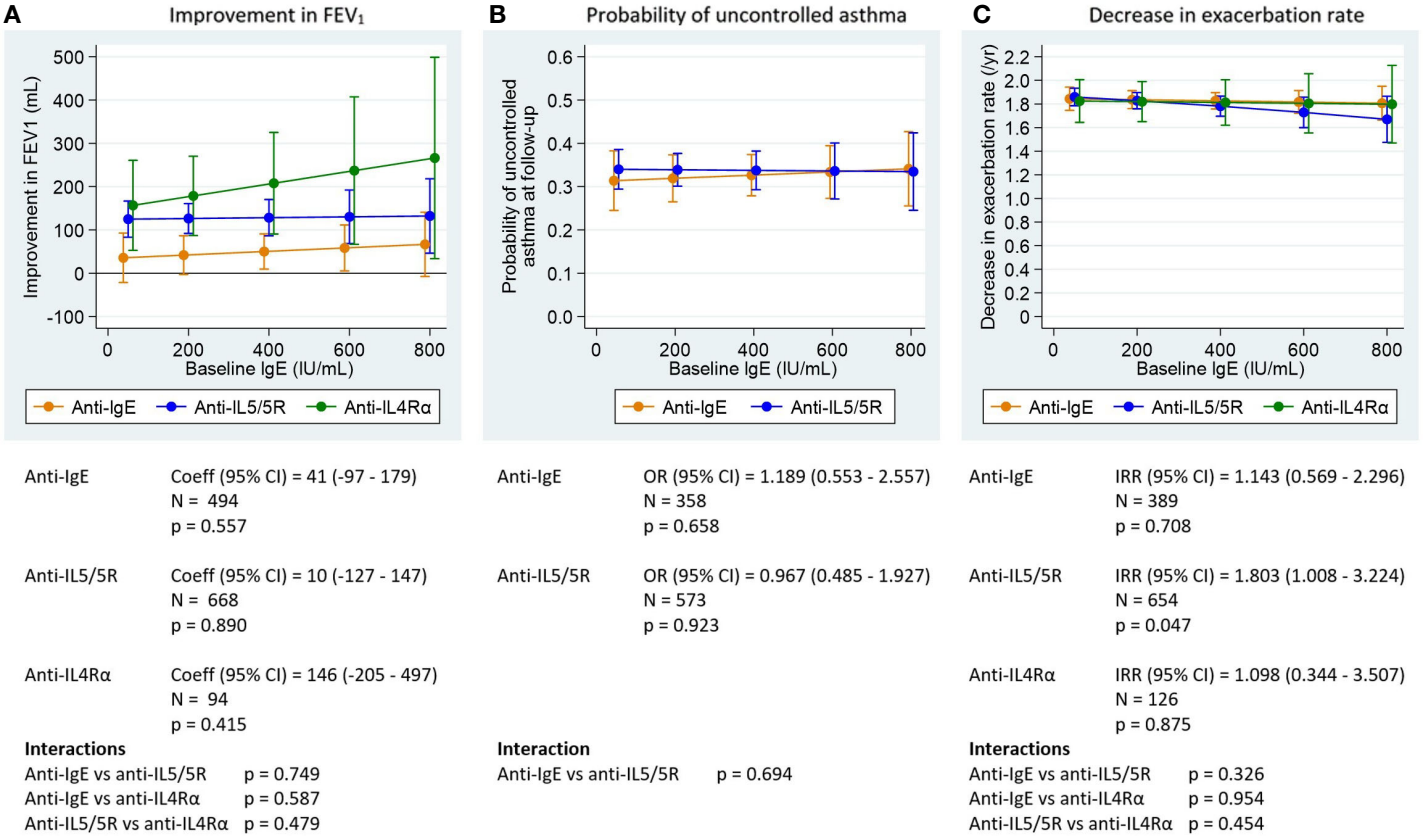
Association between pre-biologic IgE and post-biologic asthma outcomes by biologic class, adjusted for baseline for each outcome. FEV_1_, post-bronchodilator forced expiratory volume in one second; IgE, immunoglobulin E. Graphs show point estimates from the regression models for selected values of the biomarkers. **(A)** change in FEV_1_ for a patient with baseline FEV_1_ = 2.1 L (mean baseline FEV_1_ for the biologic patients in ISAR), **(B)** asthma control at follow-up for a population with 68% uncontrolled asthma at baseline (proportion for biologic patients in ISAR), **(C)** change in exacerbation rate for a patient with 2.2 exacerbations per year at baseline (mean baseline exacerbation rate for the biologic patients in ISAR). Asthma control assessed using Global Initiative for Asthma (GINA) 2020 control categories (30). For countries providing Asthma Control Questionnaire (ACQ) or Asthma Control Test (ACT) scores to rate asthma control instead of GINA control categories, conversions were performed as follows: mean ACQ score ≤0.75 =well-controlled, mean ACQ score >0.75 to <1.5 = partly controlled, mean ACQ score ≥1.5 = uncontrolled; total ACT score >19 = well-controlled, total ACT score >15 to ≤19 = partly controlled, total ACT score ≤15 = uncontrolled. Coefficients, odds ratios, and incidence rate ratios are the estimated change in the outcome per 1000 cells/µL (BEC), per 100 ppb (FeNO) or per 1000 IU/mL (IgE). P-values are for tests of association between the outcomes after treatment and baseline levels of the biomarkers (adjusted for baseline level of the outcome). Interaction tests are for comparisons of the slope coefficients for the different biologic classes, estimated by the model.

All asthma outcomes assessed improved with biologic treatment; the estimated probability of having uncontrolled asthma was approximately 0.3, and the exacerbation rate decreased by approximately 2 per year post-biologic. Pre-biologic biomarker concentrations were associated with post-biologic outcome, but this association varied by biologic class and asthma outcome assessed (**Figures 1**-**3**).

#### Association between pre-biologic BEC and post-biologic outcomes

Greater post-biologic improvement in FEV_1_ was observed in those patients with higher pre-biologic BEC for both anti-IgE and anti-IL5/5R therapies (**Figure 1A**). FEV_1_ improved by 27 mL (post- vs pre–anti-IgE therapy) for those with a mean baseline BEC of 250 cells/µL, increasing to 178 mL for those with a baseline BEC of 1000 cells/µL. Patients treated with an anti-IL5/5R therapy experienced a 43-216 mL increase in FEV_1_ along the same BEC gradient. A trend towards greater post-biologic improvement in FEV_1_ (129-250 mL) was noted for patients treated with anti-IL4Rα therapy, as pre-biologic BEC increased from 250-1,000 cells/µL (**Supplementary Table 5A**). Greater baseline BEC was also associated with a lower probability of uncontrolled asthma post-biologic, but only for patients treated with anti-IL5/5R therapy (**Figure 1B**). The probability of uncontrolled asthma when treated with anti-IL5/5R therapy was reduced from 0.42 (95% CI: 0.36, 0.48) at BEC of 50 cells/µL to 0.24 (95% CI: 0.19, 0.30) at BEC 1000 cells/µL, but remained relatively constant in the anti-IgE group, ranging from 0.35 to 0.32 over the same BEC range (**Figure 1B**; **Supplementary Table 6A**). No association was found between pre-biologic BEC and pre-to-post change in exacerbation rate for any biologic class (**Figure 1C**; **Supplementary Table 7A**).

#### Association between pre-biologic FeNO and post-biologic outcomes

Greater pre-biologic FeNO concentrations were also associated with greater FEV_1_ improvement for both anti-IgE and anti-IL5/5R therapies (**Figure 2A**). A relationship in the same direction was seen for patients treated with anti-IL4Rα therapy, but was not statistically significant. Mean FEV_1_ improved by 41 to 274 mL in the anti-IgE group along a 25-100 ppb FeNO gradient and from 69 to 207 mL and 148 to 224 mL in the anti-IL5/5R and anti-IL4Rα groups, respectively, along the same gradient (**Supplementary Table 5B**). There was no association between pre-biologic FeNO concentration and probability of uncontrolled asthma for any biologic class assessed (**Figure 2B**; **Supplementary Table 6B**). There was also no association between pre-biologic FeNO concentrations and exacerbation rate reduction for those treated with anti-IgE or anti-IL5/5R therapies (**Figure 2C**; **Supplementary Table 7B**). Although greater pre-biologic FeNO concentrations appeared to be associated with less exacerbation rate reduction for patients treated with anti-IL4Rα therapy (**Figure 2C**), the results should be interpreted with caution because small patient numbers resulted in a large range of error particularly at the higher FeNO range.

#### Association between pre-biologic IgE and post-biologic treatment outcomes

Pre-biologic IgE was a poor predictor of subsequent pre-to-post biologic change for all outcomes assessed, irrespective of biologic class (**Figures 3A–C**; **Supplementary Tables 5C**-**7C**)

#### Additional subgroup analyses

**Figure 4:**
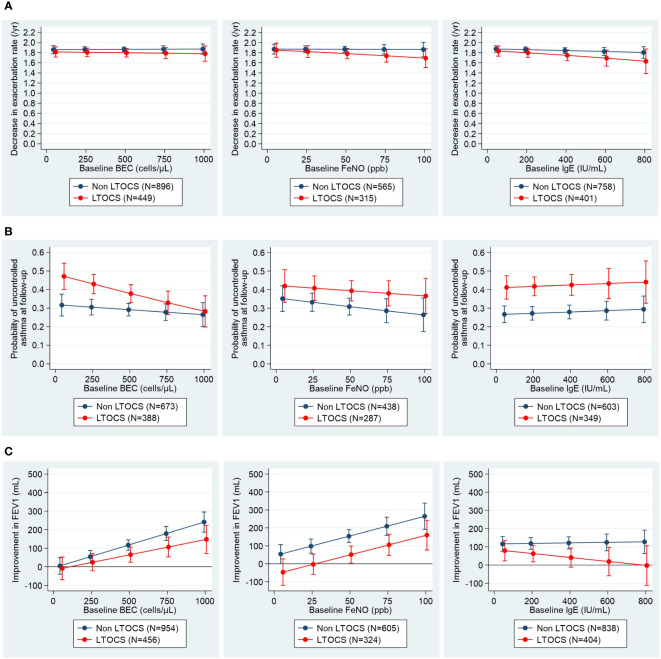
Associations between pre-biologic biomarker levels and post-biologic **(A)** exacerbation rate, **(B)** asthma control and **(C)** FEV_1_ stratified by LTOCS use. Asthma control assessed using Global Initiative for Asthma (GINA) 2020 control categories (30). For countries providing Asthma Control Questionnaire (ACQ) or Asthma Control Test (ACT) scores to rate asthma control instead of GINA control categories, conversions were performed as follows: mean ACQ score ≤0.75 =well-controlled, mean ACQ score >0.75 to <1.5 = partly controlled, mean ACQ score ≥1.5 = uncontrolled; total ACT score >19 = well-controlled, total ACT score >15 to ≤19 = partly controlled, total ACT score ≤15 = uncontrolled. BEC, blood eosinophil count; FeNO, fractional exhaled nitric oxide; FEV_1_, post-bronchodilator forced expiratory volume in one second; IgE, immunoglobulin E; LTOCS, long-term oral corticosteroid; ppb, parts per billion.

Associations between pre-biologic biomarkers and the post-biologic exacerbation and control outcomes were relatively flat irrespective of LTOCS use at biologic initiation. However, post-biologic exacerbation rate reduction tended to be better in those without LTOCS use (**Figure 4A**), patients without allergies, and for those aged ≥18 years at asthma onset (**Supplementary Figure 6**) for all biomarkers assessed. The probability of uncontrolled asthma at follow-up was also generally lower for those patients without evidence of LTOCS use at biologic initiation (**Figure 4B**), as well as those with adult-onset asthma (**Supplementary Figure 7**) for all biomarkers. By contrast, pre-biologic BEC and FeNO were generally predictive of an improved biologic lung function response in all sub-groups listed above, and irrespective of baseline exacerbation rates, with a greater improvement in FEV_1_ noted in those patients not taking LTOCS at biologic initiation and for those with adult-onset asthma (**Figure 4C**; **Supplementary Figure 8**).

#### Associations of multiple pre-biologic biomarkers and pre- to post-biologic change in asthma outcomes

**Figure 5:**
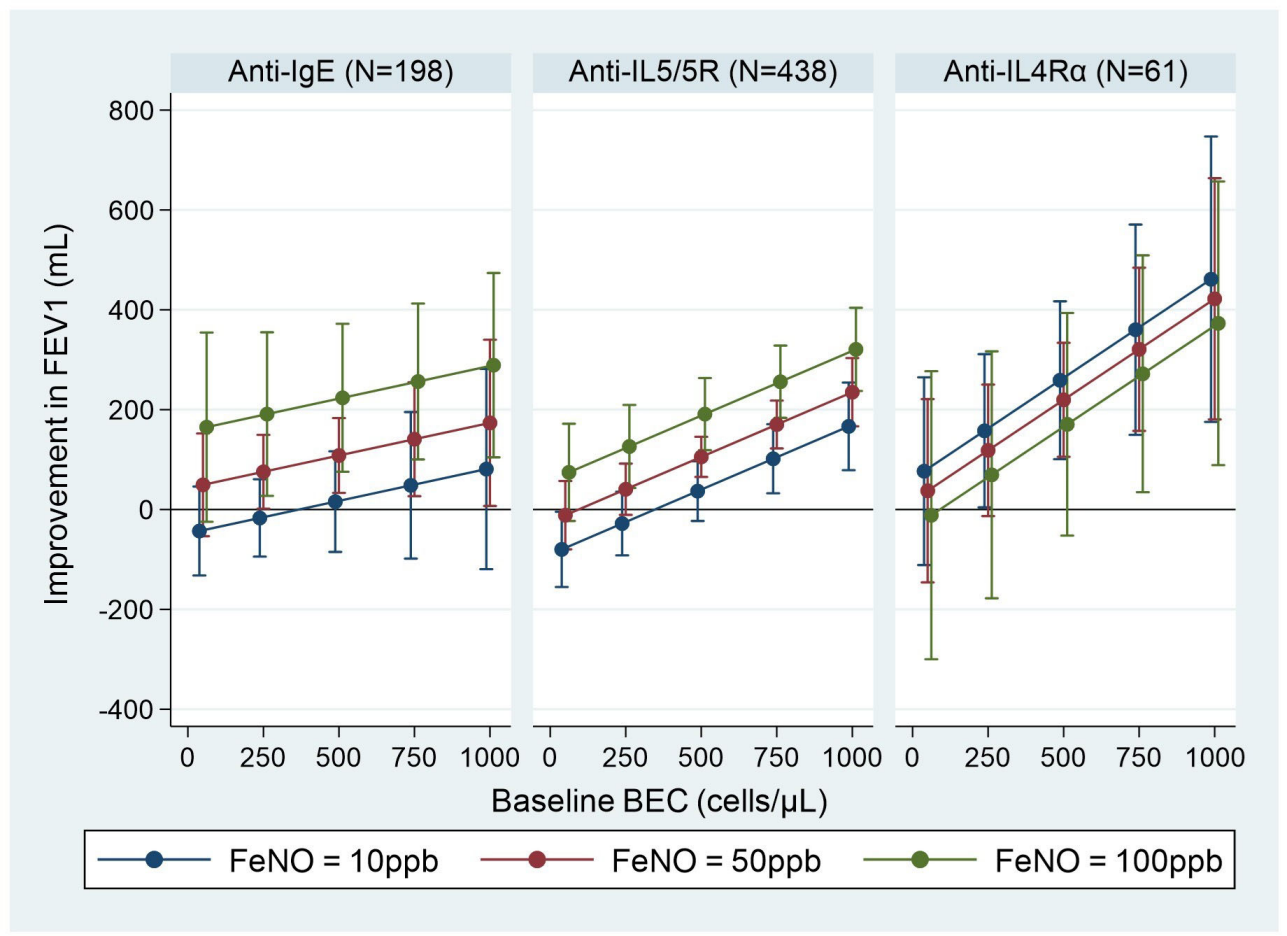
Association between pre-biologic BEC + FeNO and post biologic improvement in FEV_1_ by biologic class. BEC, blood eosinophil count; FeNO, fractional exhaled nitric oxide; FEV_1_, post-bronchodilator forced expiratory volume in one second. Graphs show point estimates (95% CI) of improvement in FEV_1_ for selected values of the biomarkers from the regression model including both baseline BEC and FeNO. Improvement in FEV_1_ is estimated for a patient with baseline FEV_1_ = 2.1 L (mean baseline FEV_1_ for the biologic patients in ISAR).

The addition of BEC + FeNO marginally improved the prediction of post-biologic FEV_1_ increase (adjusted R^2^: 0.751), compared to BEC (adjusted R^2^: 0.747) or FeNO alone (adjusted R^2^: 0.743) (p=0.005 and <0.001, respectively; **Table 3**). However, prediction of post-biologic FEV_1_ improvement was not further improved by the addition of IgE, including all three biomarkers (i.e., BEC + FeNO + IgE; adjusted R^2^: 0.750; p=0.791 vs BEC + FeNO). The estimated magnitude of BEC and FeNO effects on post-biologic improvement in lung function, when both were included in the same model is presented in **Figure 5**. We noted a positive linear relationship for BEC and FeNO for each biologic class, with FeNO appearing to have the least effect in the anti-IL4Rα group (i.e. less separation of the lines). Stratifying mean change in lung function by pre-biologic BEC + FeNO categories revealed a post-biologic change (SD) in FEV_1_, which ranged from -48 (304) mL when both BEC and FeNO were low (i.e. <150 cells/µl and <25 ppb, respectively) to 276 (506) mL when both BEC and FeNO were high (i.e. >300 cells/µL and >50 ppb, respectively) (**Supplementary Table 8A**; **Supplementary Figure 9**). A similar pattern was noted by biologic class, with post-biologic lung function improving along a gradient from BEC + FeNO low to BEC + FeNO high, although patient numbers in each category were low (**Supplementary Table 8B**).

**Table 3:** Effect of using combined biomarkers to predict outcomes after biologic treatment.

		FEV_1_		Asthma control		Exacerbations	
Biomarkers in model	Biomarkers in comparator model	Adjusted R^2^ [1]	P-value compared to comparator model	% correctly classified [2]	P-value compared to comparator model	Mean absolute error [3]	P-value compared to comparator model
None (baseline outcome only)		0.737		62.4		0.622	
BEC	None	0.747*	<0.001	62.4	0.061	0.617	0.782
FeNO	None	0.743	<0.001	63.1*	0.145	0.612*	0.294
IgE	None	0.736	0.732	62.4	0.660	0.619	0.167
BEC & FeNO	BEC	0.751**	0.005	64.3**	0.292	0.601	0.217
	FeNO		<0.001		0.123		0.613
BEC & IgE	BEC	0.747	0.860	61.3	0.595	0.614	0.159
	IgE		<0.001		0.055		0.755
FeNO & IgE	FeNO	0.743	0.718	63.9	0.626	0.607	0.087
	IgE		<0.001		0.137		0.157
BEC & FeNO & IgE	BEC	0.750	0.029	64.3	0.464	0.597**	0.089
	FeNO		<0.001		0.256		0.215
	IgE		<0.001		0.079		0.323
	BEC & FeNO		0.791		0.569		0.089
	BEC & IgE		0.004		0.279		0.122
	FeNO & IgE		<0.001		0.112		0.622

All models were adjusted for baseline level of the outcome.

P-values are for likelihood ratio tests comparing the model including the stated biomarkers with the comparator model, to illustrate the effects of including additional biomarkers on the predictive accuracy of the model. Significant P-values indicate an improvement in the model predictions as a result of including the additional biomarkers (vs. the comparator model).

Based on the criteria shown, within each outcome: *indicates the best single biomarker model, **indicates the best multi-biomarker model for each outcome.

1. Adjusted R^2^ is a measure of the proportion of variation in FEV_1_ explained by the model.

2. Predicted values from the model were classified as correct if predicted probability > 0.5% and actual outcome was uncontrolled asthma or predicted probability < 0.5 and actual outcome was not uncontrolled asthma.

3. For each patient, the actual follow-up exacerbation rate was subtracted from the exacerbation rate predicted by the model, ignoring the direction of the error. The mean of these absolute errors gives an indication of how well the model could predict follow-up exacerbation rates.

FeNO, fractional exhaled nitric oxide; FEV_1_, post-bronchodilator forced expiratory volume in one second; IgE, immunoglobulin E.

## Discussion

For efficient and cost-effective adoption of targeted treatment options in daily clinical practice, clinicians need point-of-care, well-defined, and reliable biomarkers or a combination of biomarkers to support them in identifying phenotypes and endotypes of asthma more likely to respond to biologic therapy (40). The real-life nature of our study was designed to directly inform that need, investigating pre-to-post biologic change in asthma outcomes along a gradient for three biomarkers used routinely in clinical practice (i.e., BEC, FeNO, and IgE), for three biologic classes (i.e., anti-IgE, anti-IL5/5R and anti-IL4Rα), and across 3 asthma outcomes (i.e., exacerbation rate, asthma control, and lung function). We found that individual biomarkers at their highest pre-biologic concentrations were associated with greater pre-to-post biologic change in some outcomes assessed; the strength of the association was both asthma outcome- and biologic class-specific. Overall, it appeared that pre-biologic biomarkers were not strongly associated with within-group decreases in exacerbation rate following biologic treatment. However, pre-biologic BEC and FeNO were strongly associated with lung function improvement for both anti-IgE and anti-IL5/5R therapies, with BEC also associated with improved asthma control for patients treated with anti-IL5/5R therapies. Use of biomarker combinations provided a small improvement in prediction of biologic-associated effectiveness, most notably the effectiveness on FEV_1_, and was unlikely to be of clinical significance for predicting any of the three outcomes studied.

GINA 2023 lists high BEC as a predictor of asthma response to anti-IgE, anti-IL5/5R, anti-IL4Rα, and anti-TSLP therapies for those with severe asthma and exacerbations in the last year (41) based on reported evidence of a strong association between pre-biologic BEC and post-biologic improvement in exacerbations (8, 42). Our results do not contradict that position, but rather represent a consequence of assessing the relationship between pre-biologic biomarker concentration and exacerbations in a different way (i.e., we observed a clinically meaningful reduction in exacerbation rate for anti-IgE, anti-IL5/5R, and anti-IL4Rα therapies, both at the high and low end of the concentration range for each biomarker assessed, but this reduction was relative to pre-biologic status, not compared to control). Indeed, others have shown the same relatively flat association of BEC concentration with pre-to-post biologic-associated exacerbation rate reduction (8, 41, 42). Our findings also may have been influenced by selection bias, i.e. how patients were selected for subsequent biologic treatment. Indeed, an additional *post-hoc* analysis again found little association between pre-biologic BEC and baseline exacerbation rate for patients who subsequently initiated biologics (most likely due to high BEC, a requirement for treatment with an anti-IL5/5R therapy), but a clear association was observed for those who were not started on any biologic (**Supplementary Figure 10**). We also included a much wider range of biomarker values (and wider gradient of biologic response) and our results may also have been influenced by noise from non-pathophysiologic factors in real-world patients not observed in tightly controlled RCT populations in which signal outweighs noise.

Higher pre-biologic BEC concentrations were, however, associated with greater improvements in lung function (anti-IgE and anti-IL5/5R therapies) and better asthma control (anti-IL5/5R therapies), a finding supported by others, who showed that sputum T2 markers (e.g. eosinophil count) seemed to be potentially predictive of super-response and remission after anti-IL5/5R therapy in a cohort of patients with severe eosinophilic asthma (43, 44). Improvement in lung function noted in the current study was particularly marked—approximately 100 mL and 200 mL for those with a BEC of 500 cells/µL and 1000 cells/µL, respectively. Even greater lung function improvements have recently been reported with increasing BEC following 52-week treatment with benralizumab: 1,081 mL FEV_1_ improvement in those with BEC >500 cells/µL compared to a 690 mL improvement in those with BEC ≤500 cells/µL, albeit in a small cohort of 18 patients with marked lung function impairment (mean ppFEV_1_: 56.7%) (19). Dupilumab, on the other hand, was found to increase lung function in patients with uncontrolled persistent asthma irrespective of BEC, increasing FEV_1_ by up to 430 mL in those with BEC ≥300 cells/µL (20). The ability to accurately predict which patients will experience a clinically relevant improvement in lung function when treated with biologics has important implications for earlier therapeutic intervention before permanent deterioration in lung function has occurred, when asthma is high-risk rather than severe. Indeed, as lung function in patients with severe asthma is quite heterogenous, it may be a more precise (or useful) tool to gauge biologic response than exacerbation rate (which is consistently high) or control (which is consistently poor) in this asthma population (29). Although Casale and colleagues found in the PROSPERO study that baseline BEC was associated with anti-IgE–induced improvement in asthma control and lung function, the magnitude of this improvement was not considered to be clinically relevant by the investigators and was not explored along a biomarker concentration gradient (45).

The current study also found a relationship between increasing pre-biologic FeNO levels and improved post-biologic outcome, but only for lung function, presumably because increased FeNO levels are associated with poor lung function and accelerated decline in lung function (46). GINA 2023 lists elevated baseline FeNO as predictive of response to anti-IgE, anti-IL4R, and anti-TSLP therapies, but not to anti-IL5/5R therapies (41). Our findings and those of others support this position for anti-IgE therapy (47, 48); however, we also found an association between pre-biologic FeNO and lung function improvement post–anti-IL5/5R treatment. This was not, however, apparent for anti-IL4Rα therapy, likely due to the small numbers of patients in this group and the large confidence intervals, particularly at the higher end of the FeNO concentration range—although interestingly, FeNO levels were markedly reduced with anti-IL4Rα therapy (**Supplementary Figure 4**). A recent large Phase 3 study by Pavord and colleagues found that baseline FeNO independently predicted dupilumab-associated lung function improvement in patients with moderate-to-severe asthma, with a 350 mL FEV_1_ improvement relative to placebo seen in those with FeNO >50 ppb compared to 40 mL for those with FeNO <25 ppb (12). Indeed, the European Academy of Allergy Asthma & Clinical Immunology (EAACI) position paper on clinically applicable biomarkers for asthma cites FeNO as the best biomarker to guide anti-IL4Rα-targeted (endotypic) therapy, which was evaluated following the SAVED approach (18). It should be noted that although it is a reproducible, easily measured, non-invasive biomarker, FeNO concentrations can fluctuate within days and may be affected by smoking history, atopy, adherence to inhaled corticosteroids, and high nitrite diet (18, 49–51).

We found no association between serum IgE concentrations and anti-IgE effectiveness. Consistent correlations between anti-IgE treatment response and baseline total serum IgE or antigen-specific IgE concentrations are lacking (52, 53), although recently use of cumulative IgE levels has been suggested (54). When patients were stratified *post-hoc* by T2 biomarkers, exacerbations were reduced to a greater degree in those with the presence of both modestly elevated IgE and T2 biomarkers, despite similar IgE concentrations (55). The EAACI position paper states that in severe allergic asthma, serum total IgE is useful in identifying patients who could benefit from anti-IgE therapy, but it cannot predict the degree of response after treatment (18). In patients with concomitant high eosinophil levels whose asthma remains uncontrolled, switching to an anti-eosinophilic treatment (i.e., anti-IL5/5R) might be a good option (18).

We found little added value in using composite biomarkers to predict biologic effectiveness. Such an effect may be apparent in a broader population with more heterogeneous asthma control and exacerbation rates, at least within countries. For example, we have previously found a greater pre-to-post biologic effect in patients with severe asthma and comorbid chronic rhinosinusitis with or without nasal polyps, which is associated with high BEC and FeNO levels (56). Lack of an association in our study may be due to poor correlation of biomarkers with each other, particularly for FeNO/IgE and BEC/IgE. One would expect highly correlated biomarkers to tell us the same thing, so a low correlation could be expected to make multiple biomarkers more useful, although this was something we did not find in our analysis. We also examined the effect of pre-biologic biomarker levels within group (i.e., pre-to-post) rather than versus placebo comparisons—hence the need for very large patient numbers to show within-arm difference—and acknowledge that multiple biomarkers will only perform better if they reflect or define a different entity that is capable of responding to treatment.

The RASP (Refractory Asthma Stratification Programme)-UK study group is examining the predictive value of using serum periostin, FeNO, and BEC as a composite biomarker to predict exacerbation risk, with preliminary results indicating that use of the three biomarkers in a ‘composite’ score further differentiated patients on the basis of exacerbation rate (57). U-BIOPRED (Unbiased biomarkers in Prediction of Respiratory Disease Outcomes) and SARP (Severe Asthma Research Program) are also seeking to better understand the prognostic value of individual biomarkers, their relationship to each other, and the prognostic value of using composite biomarkers or biomarker patterns (58).

Strengths of our study include inclusion of a large, heterogenous, adult severe asthma population, encompassing patients from 23 countries and representative of the general severe asthma population. The study was statistically powered to investigate differences across multiple outcome domains (i.e., exacerbation rate, lung function, and asthma control) along a biomarker concentration gradient (not simply dichotomized as high vs low) in a real-life setting. All analyses included at least 90 patients within each of the groups. This would have given us at least 80% power to observe an increase of ≥393 mL in FEV_1_, an incidence rate ratio of ≥1.85 for exacerbation rates, and an odds ratio ≤0.30 for uncontrolled asthma at follow-up per increment of 1,000 cells/µL BEC.

Limitations of our study included the small sample sizes for those treated with anti-IL4Rα therapy and those with values for all three biomarkers, and small numbers of patients with very high biomarker values, which could affect effect estimates at the highest end of the biomarker concentration spectrum. Timing of biomarker measurements and assessment of outcomes also varied considerably between patients. In common with other real-life studies of similar design without a comparator group, change in asthma outcomes may have been due to regression to the mean, subject to time-varying confounding. Furthermore, although we compared across biologic classes (i.e. anti-IgE, anti-IL5/5R and anti-IL4Rα), the remit of ISAR precludes direct comparison of biologics within class. Finally, 883 of 4,895 (18%) patients eligible for inclusion in the analysis of outcomes were excluded due to biologic switching. This is a potential source of bias; however, inclusion of those who switched biologic therapies may also have introduced bias. Further work on timing of biomarker assessment is warranted (i.e., highest pre-biologic biomarker concentration or value closest to biologic initiation), how biomarkers and combinations of biomarkers are associated with outcomes in those who switch biologics, and the identification of other biomarkers that may predict better response to biologics across a range of outcomes is warranted.

Our results provide a better understanding of pre-biologic biomarkers associated with better specific outcomes when treated with biologic therapy in a real-life setting and may be useful for clinicians when deciding between anti-IgE, anti-IL5/5R, and anti-IL4Rα biologic therapy options. The ability of both BEC and FeNO to predict biologic-associated improvement in FEV_1_ may encourage earlier intervention in patients at risk of accelerated lung function decline and promotes the use of lung function as a sensitive outcome to both predict and assess biologic effectiveness. The complexity of the asthma endotype requires going beyond the use of a ‘one size fits all’ composite biomarker predictive tool and instead opting for a more personalized approach matching biomarker(s), biologic, and asthma outcome.

## Data availability statement

The dataset supporting the conclusions of this article was derived from the International Severe Asthma Registry (ISAR). The authors do not have permission to give public access to the study dataset; researchers may request access to ISAR data for their own purposes. ISAR research requests and proposals can be made via the ISAR website (https://isaregistries.org/research-proposal-requests/) or via the enquiries email to info@isaregistries.org. In line with ISAR governance restrictions, sharing individual deidentified participant data is subject to the consent of the ISAR steering committee in accordance with patient consent, patient confidentiality and ethical considerations. The study documents (protocol, statistical analysis plan, clinical study report) will be made available in accordance with the criteria of the European Network of Centres for Pharmacoepidemiology and Pharmacovigilance (EUPAS38128). Proposals should be directed to info@isaregistries.org; to gain access, if approved by the regulatory boards, data requestors will need to sign a data access agreement.

## Ethics statement

This study was designed, implemented, and reported in compliance with the European Network Centres for Pharmacoepidemiology and Pharmacovigilance Code of Conduct (EMA 2014; EUPAS43806) and with all applicable local and international laws and regulation. Registration of the ISAR database with the European Union Electronic Register of Post-Authorization studies was also undertaken (ENCEPP/DSPP/23720). ISAR has ethical approval from the Anonymised Data Ethics Protocols and Transparency (ADEPT) committee (ADEPT0218). Governance was provided by The Anonymous Data Ethics Protocols and Transparency (ADEPT) committee (registration number: ADEPT1621). All data collection sites in the International Severe Asthma Registry (ISAR) have obtained regulatory agreement in compliance with specific data transfer laws, country-specific legislation, and relevant ethical boards and organizations.

## Author contributions

CP: Writing – original draft, Writing – review & editing. JT: Writing – original draft, Writing – review & editing. CB: Writing – original draft, Writing – review & editing. GCC: Writing – original draft, Writing – review & editing. GK: Writing – original draft, Writing – review & editing. DL: Writing – original draft, Writing – review & editing. TNT: Writing – original draft, Writing – review & editing. RA-L: Writing – original draft, Writing – review & editing. SB-A: Writing – original draft, Writing – review & editing. JB: Writing – original draft, Writing – review & editing. MH: Writing – original draft, Writing – review & editing. KK: Writing – original draft, Writing – review & editing. NP: Writing – original draft, Writing – review & editing. PEP: Writing – original draft, Writing – review & editing. TP: Writing – original draft, Writing – review & editing. CR: Writing – original draft, Writing – review & editing. MS: Writing – original draft, Writing – review & editing. M-JT: Writing – original draft, Writing – review & editing. CU: Writing – original draft, Writing – review & editing. MA-A: Writing – original draft, Writing – review & editing. AA: Writing – original draft, Writing – review & editing. ABe: Writing – original draft, Writing – review & editing. LBu: Writing – original draft, Writing – review & editing. VC: Writing – original draft, Writing – review & editing. BC: Writing – original draft, Writing – review & editing. KF: Writing – original draft, Writing – review & editing. SH: Writing – original draft, Writing – review & editing. LH: Writing – original draft, Writing – review & editing. RH: Writing – original draft, Writing – review & editing. PK: Writing – original draft, Writing – review & editing. RM: Writing – original draft, Writing – review & editing. TN: Writing – original draft, Writing – review & editing. LP: Writing – original draft, Writing – review & editing. DC: Writing – original draft, Writing – review & editing. FS: Writing – original draft, Writing – review & editing. MW: Writing – original draft, Writing – review & editing. RA: Writing – original draft, Writing – review & editing. ABo: Writing – original draft, Writing – review & editing. GB: Writing – original draft, Writing – review & editing. WC: Writing – original draft, Writing – review & editing. LC: Writing – original draft, Writing – review & editing. ED: Writing – original draft, Writing – review & editing. JF: Writing – original draft, Writing – review & editing. FH: Writing – original draft, Writing – review & editing. DJ: Writing – original draft, Writing – review & editing. RK: Writing – original draft, Writing – review & editing. BK: Writing – original draft, Writing – review & editing. MK: Writing – original draft, Writing – review & editing. AŁ: Writing – original draft, Writing – review & editing. LL: Writing – original draft, Writing – review & editing. ML: Writing – original draft, Writing – review & editing. BM: Writing – original draft, Writing – review & editing. NM: Writing – original draft, Writing – review & editing. AM-G: Writing – original draft, Writing – review & editing. PHwP: Writing – original draft, Writing – review & editing. AP: Writing – original draft, Writing – review & editing. PHP: Writing – original draft, Writing – review & editing. LP-d-L: Writing – original draft, Writing – review & editing. MP: Writing – original draft, Writing – review & editing. LR: Writing – original draft, Writing – review & editing. BR-C: Writing – original draft, Writing – review & editing. IS: Writing – original draft, Writing – review & editing. TRT: Writing – original draft, Writing – review & editing. CT-D: Writing – original draft, Writing – review & editing. EW: Writing – original draft, Writing – review & editing. MZ: Writing – original draft, Writing – review & editing. JA: Writing – original draft, Writing – review & editing. KA: Writing – original draft, Writing – review & editing. RC: Writing – original draft, Writing – review & editing. PG: Writing – original draft, Writing – review & editing. EH: Writing – original draft, Writing – review & editing. JM: Writing – original draft, Writing – review & editing. SN: Writing – original draft, Writing – review & editing. D-WP: Writing – original draft, Writing – review & editing. FP: Writing – original draft, Writing – review & editing. SS: Writing – original draft, Writing – review & editing. C-CS: Writing – original draft, Writing – review & editing. CS: Writing – original draft, Writing – review & editing. CTai: Writing – original draft, Writing – review & editing. TLT: Writing – original draft, Writing – review & editing. LBj: Writing – original draft, Writing – review & editing. GWC: Writing – original draft, Writing – review & editing. TI: Writing – original draft, Writing – review & editing. LJ-M: Writing – original draft, Writing – review & editing. CTau: Writing – original draft, Writing – review & editing. LBr: Writing – original draft, Writing – review & editing. DP: Writing – original draft, Writing – review & editing.

## Supplementary material

Supplementary Table 1 characteristics of patients included for assessment of biomarker distribution and biomarker correlation analyses.

Supplementary Table 2 Changes in biomarker concentration compared with baseline at different times post-biologic initiation.

Supplementary Table 3 Changes in FeNO concentration compared with baseline at different times post-biologic initiation, stratified by baseline (highest pre-biologic) FeNO measurement.

Supplementary Table 4 correlation of pre-biologic biomarkers taken with 7 days of each other stratified by LTOCS use.

Supplementary Table 5 Point estimates (with 95% CI) for FEV_1_ from the regression models are shown for selected levels of **(A)** BEC, **(B)** FeNO and **(C)** IgE.

Supplementary Table 6 Point estimates (with 95% CI) for probability of uncontrolled asthma from the regression models for selected levels of **(A)** BEC, **(B)** FeNO and **(C)** IgE concentrations.

Supplementary Table 7 Point estimates (with 95% CI) for exacerbations from the regression models for selected levels of **(A)** BEC, **(B)** FeNO and **(C)** IgE concentration.

Supplementary Table 8
**(A)** Pre-to-post biologic change in asthma outcome stratified by BEC and FeNO level: biologics overall **(B)** Pre-to-post biologic change in asthma outcome stratified by BEC and FeNO level: by biologic class.

Supplementary Figure 1 Study design.

Supplementary Figure 2 subject disposition.

Supplementary Figure 3 Distribution of pre-biologic biomarkers (highest concentration) among patients enrolled in ISAR for all patients and according to LTOCS use at baseline.

Supplementary Figure 4 Median biomarker changes compared with baseline, at different times after initiation of biologic therapy.

Supplementary Figure 5 Correlation of pre-biologic biomarkers taken within 7 days of each other among patients enrolled in ISAR.

Supplementary Figure 6 Associations between post-biologic decrease in exacerbations and pre-biologic biomarker levels by subgroups.

Supplementary Figure 7 Associations between post-biologic probability of uncontrolled asthma and pre-biologic biomarker levels by subgroups.

Supplementary Figure 8 Associations between post-biologic improvement in FEV_1_ and pre-biologic biomarker levels by subgroups.

Supplementary Figure 9 Pre-to-post biologic change in FEV_1_ (mL) according to BEC + FeNO categories.

Supplementary Figure 10 Association between baseline BEC and baseline exacerbation rate for patients, who **(A)** subsequently initiated and **(B)** did not initiate a biologic.
