# Supplementary material for: Association between pre-biologic T2-biomarker combinations and response to biologics in patients with severe asthma
Source: Front Immunol. 2024 Apr 19;15:1361891. doi: 10.3389/fimmu.2024.1361891 (PMC11070939; doi:10.3389/fimmu.2024.1361891)
Supplement: Supplementary Table 1 — characteristics of patients included for assessment of biomarker distribution and biomarker correlation analyses. [file Table_1.docx]

**S-Table 1: characteristics of patients included for assessment of biomarker distribution and biomarker correlation analyses**

|  | **Non-biologic** | **Biologic** | **Total** |
| --- | --- | --- | --- |
|  | (N=6,468) | (N=4,895) | (N=11,363) |
| **Sex** | N=6,444 | N=4,896 | N=11,344 |
| Female, n (%) | 4010 (62.2) | 3043 (62.2) | 7053 (62.2) |
| **Age at index date, yrs** |  |  |  |
| Mean (SD) | 53.0 (15.0) | 52.5 (14.5) | 52.8 (14.8) |
| **Age at asthma onset, yrs** | N=2,911 | N=2,748 | N=5,659 |
| Mean (SD) | 31.2 (19.3) | 29.6 (18.4) | 30.4 (18.9) |
| **Age group at asthma onset, yrs** | N=2,911 | N=2,748 | N=5,659 |
| <18, n (%) | 852 (29.3) | 824 (30.0) | 1,676 (29.6) |
| 18-40, n (%) | 1,092 (37.5) | 1,119 (40.7) | 2,211 (39.1) |
| 41-64, n (%) | 843 (29.0) | 730 (26.6) | 1,573 (27.8) |
| 65+, n (%) | 124 (4.3) | 75 (2.7) | 199 (3.5) |
| **Duration of asthma, yrs** | N=2,911 | N=2,748 | N=5,659 |
| Mean (IQR) | 19.1 (8.6-33.0) | 19.8 (9.7-34.0) | 19.3 (9.0-33.3) |
| **Baseline BEC (cells/µL)** | N=5,823 | N=4,451 | N=10,274 |
| Median (IQR) | 300 (190-500) | 400 (200-730) | 300 (200-600) |
| **Baseline FeNO, ppb** | N=3,221 | N=2,712 | N=5,933 |
| Median (IQR) | 25.0 (14.0-49.0) | 36.0 (18.0-72.0) | 29.0 (15.0-60.0) |
| **Baseline IgE, IU/mL** | N=4,495 | N=4,032 | N=8,527 |
| Median (IQR) | 122 (37-370) | 208 (78-534) | 158 (52-457) |
| **Received LTOCS at index date** | N=6,452 | N=4,840 | N=11,292 |
| Yes, n (%) | 488 (7.6) | 1,410 (29.1) | 1898 (16.8) |
| **Ever had nasal polyps** | N=6,326 | N=4,865 | N=11,191 |
| Yes, n (%) | 846 (13.4) | 1402 (28.8) | 2248 (20.1) |
| **≥1 allergies detected by any test** | N=3,150 | N=2,944 | N=6,094 |
| Yes, n (%) | 2,044 (64.9) | 1,938 (65.8) | 3,982 (65.3) |
| **Baseline asthma control*** | N=2,860 | N=2,250 | N=5,110 |
| Well controlled, n (%) | 541 (18.9) | 271 (12.0) | 812 (15.9) |
| Partially controlled, n (%) | 731 (25.6) | 405 (18.0) | 1,136 (22.2) |
| Uncontrolled, n (%) | 1,588 (55.5) | 1,574 (70.0) | 3,162 (61.9) |
| **Baseline FEV_1_, L** | N=4,515 | N=3,822 | N=8,337 |
| Mean (SD) | 2.0 (0.8) | 2.1 (0.8) | 2.1 (0.8) |
| **Annualized exacerbation rate** | N=5,953 | N=4,128 | N=10,081 |
| Mean (SD) | 0.8 (1.6) | 2.2 (3.0) | 1.3 (2.4) |
| **Eosinophilic grade** | N=5,823 | N=4,588 | N=10,411 |
| Grade 0: Unlikely, n (%) | 723 (12.4) | 217 (4.7) | 940 (9.0) |
| Grade 1: Least likely, n (%) | 1,196 (20.5) | 310 (6.8) | 1,506 (14.5) |
| Grade 2: Likely, n (%) | 559 (9.6) | 266 (5.8) | 825 (7.9) |
| Grade 3: Most likely, n (%) | 3,345 (57.4) | 3,795 (82.7) | 7,140 (68.6) |

Abbreviations: BEC, blood eosinophil count; FeNO, fractional exhaled nitric oxide; FEV_1_, forced expiratory volume in one second; IgE, immunoglobulin E; IQR, interquartile range; LTOCS, long-term oral corticosteroids; ppb, parts per billion, SD, standard deviation

*Asthma control assessed according to GINA 2020 criteria (1), Asthma Control Test (2), or Asthma Control Questionnaire (3).
