# Supplementary material for: Association between pre-biologic T2-biomarker combinations and response to biologics in patients with severe asthma
Source: Front Immunol. 2024 Apr 19;15:1361891. doi: 10.3389/fimmu.2024.1361891 (PMC11070939; doi:10.3389/fimmu.2024.1361891)
Supplement: Supplementary Table 2 — Changes in biomarker concentration compared with baseline at different times post-biologic initiation. [file Table_2.docx]

**S-Table 2: Changes in biomarker concentration compared with baseline at different times post-biologic initiation**

Abbreviations: BEC, blood eosinophil count; FeNO, fractional exhaled nitric oxide; IgE, immunoglobulin E; IL, interleukin; IQR, inter-quartile range; ppb, parts per billion
