# Supplementary material for: Association between pre-biologic T2-biomarker combinations and response to biologics in patients with severe asthma
Source: Front Immunol. 2024 Apr 19;15:1361891. doi: 10.3389/fimmu.2024.1361891 (PMC11070939; doi:10.3389/fimmu.2024.1361891)
Supplement: Supplementary Table 3 — Changes in FeNO concentration compared with baseline at different times post-biologic initiation, stratified by baseline (highest pre-biologic) FeNO measurement. [file Table_3.docx]

**S-Table 3: Changes in FeNO concentration compared with baseline at different times post-biologic initiation, stratified by baseline (highest pre-biologic) FeNO measurement**

Abbreviations: FeNO, fractional exhaled nitric oxide; IgE, immunoglobulin E; IL, interleukin; IQR, interquartile range; ppb, parts per billion
