# Supplementary material for: Association between pre-biologic T2-biomarker combinations and response to biologics in patients with severe asthma
Source: Front Immunol. 2024 Apr 19;15:1361891. doi: 10.3389/fimmu.2024.1361891 (PMC11070939; doi:10.3389/fimmu.2024.1361891)
Supplement: Supplementary Table 4 — correlation of pre-biologic biomarkers taken with 7 days of each other stratified by LTOCS use. [file Table_4.docx]

**S-Table 4: correlation of pre-biologic biomarkers taken with 7 days of each other stratified by LTOCS use**

|  | **BEC and FeNO** | **FeNO and IgE** | **BEC and IgE** |
| --- | --- | --- | --- |
| **All patients** | N = 3099  r = 0.40  p < 0.001 | N = 2591  r = 0.16  p < 0.001 | N = 7144  r = 0.25  p < 0.001 |
| **Non-LTOCS** | N = 2686  r = 0.41  p < 0.001 | N = 2190  r = 0.18  p < 0.001 | N = 6104  r = 0.26  p < 0.001 |
| **LTOCS** | N = 410  r = 0.32  p < 0.001 | N = 399  r = 0.04  p = 0.421 | N = 1024  r = 0.19  p < 0.001 |

Correlations between log_10_ pre-biologic biomarker values taken within 7 days of each other, overall and by LTOCS use at the time of biologic initiation

Abbreviations: BEC, blood eosinophil count; FeNO, fractional exhaled nitric oxide; IgE, immunoglobulin E; LTOCS, long-term oral corticosteroids
