# Supplementary material for: Association between pre-biologic T2-biomarker combinations and response to biologics in patients with severe asthma
Source: Front Immunol. 2024 Apr 19;15:1361891. doi: 10.3389/fimmu.2024.1361891 (PMC11070939; doi:10.3389/fimmu.2024.1361891)
Supplement: Supplementary Table 5 — Point estimates (with 95% CI) for FEV1 from the regression models are shown for selected levels of (A) BEC, (B) FeNO and (C) IgE. [file Table_5.docx]

**S-Table 5: Point estimates (with 95% CI) for FEV_1_ from the regression models are shown for selected levels of (A) BEC, (B) FeNO and (C) IgE**

| A: Improvement in FEV_1_ (L) vs BEC (cells/µL) (Anti-IgE: N=512, Anti-IL5/5R: N=789; Anti-IL4Rα: N=125) | | | | |
| --- | --- | --- | --- | --- |
| Biologic | BEC | Estimated increase | [95% Conf. | Interval] |
| Anti-IgE | 50 | -0.014 | -0.069 | 0.041 |
| Anti-IL5/5R | 50 | -0.003 | -0.055 | 0.050 |
| Anti-IL4 Rα | 50 | 0.097 | -0.026 | 0.220 |
| Anti-IgE | 250 | 0.027 | -0.014 | 0.067 |
| Anti-IL5/5R | 250 | 0.043 | 0.004 | 0.083 |
| Anti-IL4 Rα | 250 | 0.129 | 0.040 | 0.218 |
| Anti-IgE | 500 | 0.077 | 0.034 | 0.120 |
| Anti-IL5/5R | 500 | 0.101 | 0.070 | 0.132 |
| Anti-IL4 Rα | 500 | 0.169 | 0.090 | 0.249 |
| Anti-IgE | 750 | 0.128 | 0.062 | 0.194 |
| Anti-IL5/5R | 750 | 0.158 | 0.122 | 0.195 |
| Anti-IL4 Rα | 750 | 0.210 | 0.096 | 0.323 |
| Anti-IgE | 1,000 | 0.178 | 0.083 | 0.274 |
| Anti-IL5/5R | 1,000 | 0.216 | 0.164 | 0.268 |
| Anti-IL4 Rα | 1,000 | 0.250 | 0.084 | 0.415 |
| B: Improvement in FEV_1_ (L) vs FeNO (ppb)  (Anti-IgE: N=254, Anti-IL5/5R: N=588; Anti-IL4Rα: N=92) | | | | |
| Biologic | FeNO | Estimated increase | [95% Conf. | Interval] |
| Anti-IgE | 5 | -0.021 | -0.098 | 0.056 |
| Anti-IL5/5R | 5 | 0.032 | -0.024 | 0.089 |
| Anti-IL4 Rα | 5 | 0.128 | -0.006 | 0.261 |
| Anti-IgE | 25 | 0.041 | -0.015 | 0.098 |
| Anti-IL5/5R | 25 | 0.069 | 0.026 | 0.112 |
| Anti-IL4 Rα | 25 | 0.148 | 0.049 | 0.247 |
| Anti-IgE | 50 | 0.119 | 0.056 | 0.182 |
| Anti-IL5/5R | 50 | 0.115 | 0.080 | 0.151 |
| Anti-IL4 Rα | 50 | 0.173 | 0.078 | 0.269 |
| Anti-IgE | 75 | 0.196 | 0.100 | 0.293 |
| Anti-IL5/5R | 75 | 0.161 | 0.117 | 0.206 |
| Anti-IL4 Rα | 75 | 0.199 | 0.063 | 0.335 |
| Anti-IgE | 100 | 0.274 | 0.135 | 0.413 |
| Anti-IL5/5R | 100 | 0.207 | 0.144 | 0.270 |
| Anti-IL4 Rα | 100 | 0.224 | 0.030 | 0.419 |
| C: Improvement in FEV_1_ (L) vs IgE (IU/mL)  (Anti-IgE: N=494, Anti-IL5/5R: N=668; Anti-IL4Rα: N=94) | | | | |
| Biologic | IgE | Estimated increase | [95% Conf. | Interval] |
| Anti-IgE | 50 | 0.036 | -0.021 | 0.093 |
| Anti-IL5/5R | 50 | 0.125 | 0.083 | 0.167 |
| Anti-IL4 Rα | 50 | 0.157 | 0.053 | 0.261 |
| Anti-IgE | 200 | 0.042 | -0.003 | 0.087 |
| Anti-IL5/5R | 200 | 0.126 | 0.092 | 0.161 |
| Anti-IL4 Rα | 200 | 0.179 | 0.087 | 0.270 |
| Anti-IgE | 400 | 0.050 | 0.010 | 0.091 |
| Anti-IL5/5R | 400 | 0.128 | 0.086 | 0.170 |
| Anti-IL4 Rα | 400 | 0.208 | 0.090 | 0.325 |
| Anti-IgE | 600 | 0.058 | 0.005 | 0.111 |
| Anti-IL5/5R | 600 | 0.130 | 0.068 | 0.192 |
| Anti-IL4 Rα | 600 | 0.237 | 0.067 | 0.407 |
| Anti-IgE | 800 | 0.067 | -0.008 | 0.141 |
| Anti-IL5/5R | 800 | 0.132 | 0.046 | 0.218 |
| Anti-IL4 Rα | 800 | 0.266 | 0.034 | 0.499 |

Abbreviations: Anti-IL4Ra, anti-interleukin 4 receptor alpha; Anti-IL5/5R, anti-interleukin 5/5 receptor; BEC, blood eosinophil count; FeNO, fractional exhaled nitric oxide; FEV_1_, forced expiratory volume in one second; IgE, immunoglobulin
