# Supplementary material for: Association between pre-biologic T2-biomarker combinations and response to biologics in patients with severe asthma
Source: Front Immunol. 2024 Apr 19;15:1361891. doi: 10.3389/fimmu.2024.1361891 (PMC11070939; doi:10.3389/fimmu.2024.1361891)
Supplement: Supplementary Table 6 — Point estimates (with 95% CI) for probability of uncontrolled asthma from the regression models for selected levels of (A) BEC, (B) FeNO and (C) IgE concentrations. [file Table_6.docx]

**S-Table 6: Point estimates (with 95% CI) for probability of uncontrolled asthma from the regression models for selected levels of (A) BEC, (B) FeNO and (C) IgE concentrations**

| A: Probability of uncontrolled asthma vs BEC (cells/µL)  (Anti-IgE: N=347, Anti-IL5/5R: N=681) | | | | |
| --- | --- | --- | --- | --- |
| Biologic | BEC | Estimated probability | [95% Conf. | Interval] |
| Anti-IgE | 50 | 0.354 | 0.282 | 0.427 |
| Anti-IL5/5R | 50 | 0.419 | 0.357 | 0.481 |
| Anti-IgE | 250 | 0.347 | 0.295 | 0.399 |
| Anti-IL5/5R | 250 | 0.379 | 0.334 | 0.423 |
| Anti-IgE | 500 | 0.339 | 0.284 | 0.393 |
| Anti-IL5/5R | 500 | 0.330 | 0.296 | 0.364 |
| Anti-IgE | 750 | 0.330 | 0.248 | 0.413 |
| Anti-IL5/5R | 750 | 0.284 | 0.242 | 0.326 |
| Anti-IgE | 1,000 | 0.322 | 0.204 | 0.440 |
| Anti-IL5/5R | 1,000 | 0.242 | 0.186 | 0.298 |
| B: Probability of uncontrolled asthma vs FeNO (ppb)  (Anti-IgE: N=186, Anti-IL5/5R: N=517) | | | | |
| Biologic | FeNO | Estimated probability | [95% Conf. | Interval] |
| Anti-IgE | 5 | 0.352 | 0.256 | 0.448 |
| Anti-IL5/5R | 5 | 0.382 | 0.316 | 0.449 |
| Anti-IgE | 25 | 0.351 | 0.281 | 0.421 |
| Anti-IL5/5R | 25 | 0.366 | 0.318 | 0.415 |
| Anti-IgE | 50 | 0.349 | 0.267 | 0.431 |
| Anti-IL5/5R | 50 | 0.347 | 0.307 | 0.387 |
| Anti-IgE | 75 | 0.347 | 0.219 | 0.475 |
| Anti-IL5/5R | 75 | 0.327 | 0.276 | 0.379 |
| Anti-IgE | 100 | 0.345 | 0.161 | 0.530 |
| Anti-IL5/5R | 100 | 0.309 | 0.236 | 0.381 |
| C: Probability of uncontrolled asthma vs IgE (IU/mL)  (Anti-IgE: N=358, Anti-IL5/5R: N=573) | | | | |
| Biologic | IgE | Estimated probability | [95% Conf. | Interval] |
| Anti-IgE | 50 | 0.314 | 0.245 | 0.383 |
| Anti-IL5/5R | 50 | 0.340 | 0.294 | 0.386 |
| Anti-IgE | 200 | 0.319 | 0.265 | 0.373 |
| Anti-IL5/5R | 200 | 0.339 | 0.301 | 0.377 |
| Anti-IgE | 400 | 0.326 | 0.279 | 0.374 |
| Anti-IL5/5R | 400 | 0.338 | 0.293 | 0.382 |
| Anti-IgE | 600 | 0.334 | 0.273 | 0.395 |
| Anti-IL5/5R | 600 | 0.336 | 0.271 | 0.401 |
| Anti-IgE | 800 | 0.341 | 0.255 | 0.427 |
| Anti-IL5/5R | 800 | 0.335 | 0.245 | 0.424 |

Abbreviations: Anti-IL5/5R, anti-interleukin 5/5 receptor; BEC, blood eosinophil count; FeNO, fractional exhaled nitric oxide; IgE, immunoglobulin

Asthma control assessed according to GINA 2020 criteria (1), Asthma Control Test (2), or Asthma Control Questionnaire (3).
