# Supplementary material for: Association between pre-biologic T2-biomarker combinations and response to biologics in patients with severe asthma
Source: Front Immunol. 2024 Apr 19;15:1361891. doi: 10.3389/fimmu.2024.1361891 (PMC11070939; doi:10.3389/fimmu.2024.1361891)
Supplement: Supplementary Table 7 — Point estimates (with 95% CI) for exacerbations from the regression models for selected levels of (A) BEC, (B) FeNO and (C) IgE concentration. [file Table_7.docx]

**S-Table 7: Point estimates (with 95% CI) for exacerbations from the regression models for selected levels of (A) BEC, (B) FeNO and (C) IgE concentration**

| A: Decrease in exacerbations (number/year) vs BEC (cells/µL)  (Anti-IgE: N=412, Anti-IL5/5R: N=772; Anti-IL4Rα: N=172) | | | | |
| --- | --- | --- | --- | --- |
| Biologic | BEC | Estimated decrease/yr | [95% Conf. | Interval] |
| Anti-IgE | 50 | 1.89 | 1.81 | 1.98 |
| Anti-IL5/5R | 50 | 1.80 | 1.69 | 1.90 |
| Anti-IL4 Rα | 50 | 1.81 | 1.61 | 2.00 |
| Anti-IgE | 250 | 1.88 | 1.81 | 1.94 |
| Anti-IL5/5R | 250 | 1.80 | 1.73 | 1.88 |
| Anti-IL4 Rα | 250 | 1.85 | 1.72 | 1.98 |
| Anti-IgE | 500 | 1.85 | 1.78 | 1.92 |
| Anti-IL5/5R | 500 | 1.81 | 1.75 | 1.87 |
| Anti-IL4 Rα | 500 | 1.90 | 1.78 | 2.01 |
| Anti-IgE | 750 | 1.82 | 1.72 | 1.93 |
| Anti-IL5/5R | 750 | 1.82 | 1.74 | 1.89 |
| Anti-IL4 Rα | 750 | 1.94 | 1.79 | 2.08 |
| Anti-IgE | 1,000 | 1.79 | 1.62 | 1.96 |
| Anti-IL5/5R | 1,000 | 1.82 | 1.72 | 1.93 |
| Anti-IL4 Rα | 1,000 | 1.97 | 1.79 | 2.15 |
| B: Decrease in exacerbations (number/year) vs FeNO (ppb)  (Anti-IgE: N=211, Anti-IL5/5R: N=559; Anti-IL4Rα: N=116) | | | | |
| Biologic | FeNO | Estimated decrease/yr | [95% Conf. | Interval] |
| Anti-IgE | 5 | 1.78 | 1.63 | 1.93 |
| Anti-IL5/5R | 5 | 1.85 | 1.74 | 1.97 |
| Anti-IL4 Rα | 5 | 2.03 | 1.91 | 2.16 |
| Anti-IgE | 25 | 1.81 | 1.71 | 1.91 |
| Anti-IL5/5R | 25 | 1.83 | 1.74 | 1.92 |
| Anti-IL4 Rα | 25 | 1.98 | 1.85 | 2.10 |
| Anti-IgE | 50 | 1.85 | 1.76 | 1.94 |
| Anti-IL5/5R | 50 | 1.81 | 1.74 | 1.89 |
| Anti-IL4 Rα | 50 | 1.88 | 1.74 | 2.02 |
| Anti-IgE | 75 | 1.88 | 1.76 | 2.01 |
| Anti-IL5/5R | 75 | 1.79 | 1.69 | 1.88 |
| Anti-IL4 Rα | 75 | 1.74 | 1.51 | 1.97 |
| Anti-IgE | 100 | 1.91 | 1.75 | 2.08 |
| Anti-IL5/5R | 100 | 1.76 | 1.62 | 1.90 |
| Anti-IL4 Rα | 100 | 1.54 | 1.08 | 2.00 |
| C: Decrease in exacerbations (number/year) vs IgE (IU/mL)  (Anti-IgE: N=389, Anti-IL5/5R: N=654; Anti-IL4Rα: N=126) | | | | |
| Biologic | IgE | Estimated decrease/yr | [95% Conf. | Interval] |
| Anti-IgE | 50 | 1.84 | 1.75 | 1.94 |
| Anti-IL5/5R | 50 | 1.86 | 1.79 | 1.93 |
| Anti-IL4 Rα | 50 | 1.83 | 1.64 | 2.01 |
| Anti-IgE | 200 | 1.84 | 1.76 | 1.91 |
| Anti-IL5/5R | 200 | 1.83 | 1.76 | 1.90 |
| Anti-IL4 Rα | 200 | 1.82 | 1.65 | 1.99 |
| Anti-IgE | 400 | 1.83 | 1.76 | 1.90 |
| Anti-IL5/5R | 400 | 1.78 | 1.70 | 1.87 |
| Anti-IL4 Rα | 400 | 1.81 | 1.62 | 2.01 |
| Anti-IgE | 600 | 1.82 | 1.72 | 1.91 |
| Anti-IL5/5R | 600 | 1.73 | 1.60 | 1.86 |
| Anti-IL4 Rα | 600 | 1.81 | 1.55 | 2.06 |
| Anti-IgE | 800 | 1.81 | 1.66 | 1.95 |
| Anti-IL5/5R | 800 | 1.67 | 1.47 | 1.87 |
| Anti-IL4 Rα | 800 | 1.80 | 1.47 | 2.13 |

Abbreviations: Anti-IL4Rα, anti-interleukin R alpha; Anti-IL5/5R, anti-interleukin 5/5 receptor; BEC, blood eosinophil count; FeNO, fractional exhaled nitric oxide; IgE, immunoglobulin
