# Supplementary material for: Association between pre-biologic T2-biomarker combinations and response to biologics in patients with severe asthma
Source: Front Immunol. 2024 Apr 19;15:1361891. doi: 10.3389/fimmu.2024.1361891 (PMC11070939; doi:10.3389/fimmu.2024.1361891)
Supplement: Supplementary Table 8 — (A) Pre-to-post biologic change in asthma outcome stratified by BEC and FeNO level: biologics overall (B) Pre-to-post biologic change in asthma outcome stratified by BEC and FeNO level: by biologic class. [file Table_8.docx]

**S-Table 8A: Pre-to-post biologic change in asthma outcome stratified by BEC and FeNO level: biologics overall**

| **BEC** | **FeNO <25 ppb** | **FeNO 25-50 ppb** | **FeNO >50 ppb** |  |
| --- | --- | --- | --- | --- |
|  |  |  |  |  |
| **Decrease in exacerbation rate /yr, mean (SD)** | | | |  |
| BEC <150 cells/µL, | N=72  1.3 (2.8) | N=39  2.7 (3.0) | N=27  2.5 (3.8) |  |
| BEC 150-300 cells/µL | N=66  2.0 (3.1) | N=50  2.2 (3.4) | N=46  2.7 (3.9) |  |
| BEC >300 cells/µL | N=93  2.4 (2.9) | N=29  2.9 (4.0) | N=146  2.3 (3.2) |  |
| **Uncontrolled asthma at follow-up**, n (%) | | | |  |
| BEC <150 cells/µL | N=65  29 (45) | N=46  12 (26) | N=31  12 (39) |  |
| BEC 150-300 cells/µL | N=72  29 (40) | N=44  15 (34) | N=39  17 (44) |  |
| BEC >300 cells/µL, | N=106  41 (39) | N=145  54 (37) | N=147  47 (32) |  |
| **Increase in FEV_1_, mL, Mean (SD)** | | | |  |
| BEC <150 cells/µL | N=69  -48 (304) | N=38  66 (412) | N=30  120 (577) |  |
| BEC 150-300 cells/µL | N=76  -20 (430) | N=52  -39 (354) | N=39  -7 (393) |  |
| BEC >300 cells/µL | N=108  74 (394) | N=134  134 (467) | N=151  276 (509) |  |

Abbreviations: Anti-IL5/5R, anti-interleukin 5/5 receptor; BEC, blood eosinophil count; FeNO, fractional exhaled nitric oxide; FEV_1_, forced expiratory volume in one second; IgE, immunoglobulin; SD: standard deviation

Asthma control assessed according to GINA 2020 criteria (1), Asthma Control Test (2), or Asthma Control Questionnaire (3).
