# Supplementary material for: Association between pre-biologic T2-biomarker combinations and response to biologics in patients with severe asthma
Source: Front Immunol. 2024 Apr 19;15:1361891. doi: 10.3389/fimmu.2024.1361891 (PMC11070939; doi:10.3389/fimmu.2024.1361891)
Supplement: Supplementary file 9 [file Table_9.docx]

**S-Table 8B: Pre-to-post biologic change in asthma outcome stratified by BEC and FeNO level: by biologic class**

| BEC | FeNO <25 ppb | | | FeNO 25-50 ppb | | | FeNO >50 ppb | | |
| --- | --- | --- | --- | --- | --- | --- | --- | --- | --- |
|  |  |  |  |  |  |  |  |  |  |
| Decrease in exacerbation rate/yr, mean (SD) | | | | | | | | | |
|  | Anti-IgE | Anti-IL5/5R | Anti-IL4Rα | Anti-IgE | Anti-IL5/5R | Anti-IL4Rα | Anti-IgE | Anti-IL5/5R | Anti-IL4Rα |
| BEC <150 cells/µL | N=33  1.2 (3.2) | N=28  1.8 (2.6) | N=11  0.4 (1.3) | N=17  2.3 (3.4) | N=18  3.5 (2.8) | N=4  0.5 (1.0) | N=7  1.1 (1.8) | N=18  3.3 (4.4) | N=2  0.0 (0.0) |
| BEC 150-300 cells/µL | N=33  1.1 (1.6) | N=21  4.1 (4.4) | N=12  0.6 (1.0) | N=15  1.0 (1.9) | N=30  3.0 (4.0) | N=5  0.7 (0.9) | N=14  2.4 (5.2) | N=23  3.8 (3.4) | N=9  0.6 (0.8) |
| BEC >300 cells/µL | N=16  2.0 (2.7) | N=68  2.6 (3.0) | N=9  1.3 (1.6) | N=20  1.3 (1.8) | N=100  3.4 (4.3) | N=9  0.7 (1.5) | N=18  2.0 (3.2) | N=111  2.7 (3.3) | N=17  0.4 (1.3) |
| Uncontrolled asthma at follow-up, n (%) | | | | | | | | | |
| BEC <150 cells/µL | N=33  16 (49) | N=28  11 (39) | N=4  2 (50) | N=20  5 (25) | N=23  7 (30) | N=3  0 (0) | N=8  3 (38) | N=21  8 (38) | N=2  1 (50) |
| BEC 150-300 cells/µL | N=38  13 (34) | N=30  13 (43) | N=4  3 (75) | N=12  4 (33) | N=30  11 (37) | N=2  0 (0) | N=9  4 (44) | N=25  12 (48) | N=5  1 (20) |
| BEC >300 cells/µL | N=23  10 (44) | N=78  31 (40) | N=5  0 (0) | N=29  11 (38) | N=106  38 (36) | N=10  5 (50) | N=19  9 (47) | N=119  37 (31) | N=9  1 (11) |
| Increase in FEV_1_, mL, mean (SD) | | | | | | | | | |
| BEC <150 cells/µL | N=38  -88 (307) | N=25  3 (301) | N=6  -3 (315) | N=16  125 (399) | N=21  31 (433) | N=1  -140 (.) | N=9  38 (886) | N=19  149 (414) | N=2  215 (431) |
| BEC 150-300 cells/µL | N=41  -24 (345) | N=25  -128 (300) | N=10  268 (805) | N=14  -149 (472) | N=33  -8 (298) | N=5  60 (309) | N=10  55 (463) | N=22  -75 (411) | N=7  114 (165) |
| BEC >300 cells/µL | N=21  11 (295) | N=76  62 (427) | N=11  278 (258) | N=27  184 (467) | N=98  109 (464) | N=9  250 (520) | N=22  280 (500) | N=119  278 (516) | N=10  234 (485) |

Abbreviations: Anti-IL5/5R, anti-interleukin 5/5 receptor; BEC, blood eosinophil count; FeNO, fractional exhaled nitric oxide; FEV_1_, forced expiratory volume in one second; IgE, immunoglobulin; SD, standard deviation

Asthma control assessed according to GINA 2020 criteria (1), Asthma Control Test (2), or Asthma Control Questionnaire (3).
