# Supplementary material for: Association between pre-biologic T2-biomarker combinations and response to biologics in patients with severe asthma
Source: Front Immunol. 2024 Apr 19;15:1361891. doi: 10.3389/fimmu.2024.1361891 (PMC11070939; doi:10.3389/fimmu.2024.1361891)
Supplement: Supplementary Figure 1 — Study design. [file Image_1.pdf]

S-Figure 1: Study design

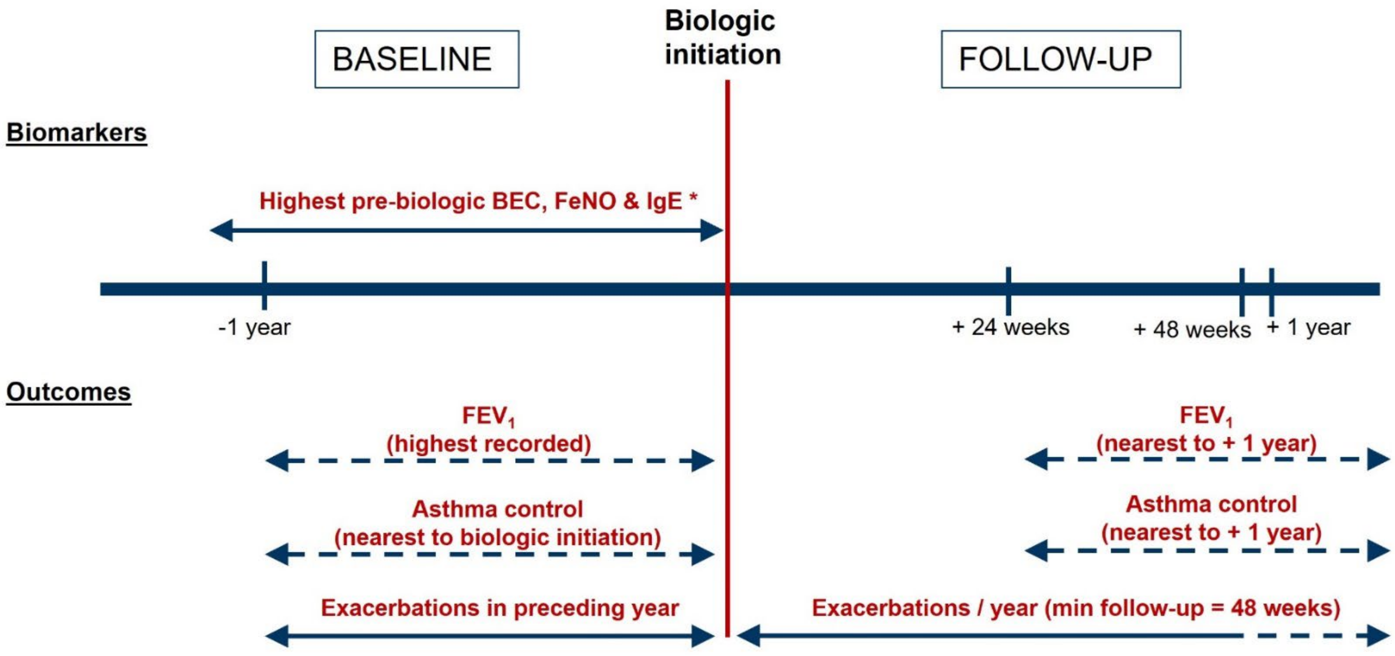

Abbreviations: BEC, blood eosinophil count; FeNO, fractional exhaled nitric oxide; FEV_1, post-bronchodilator forced expiratory volume in one second; IgE, immunoglobulin E.

*For non-biologic patients included to assess correlations between biomarkers, the highest biomarker results collected at any time were used.

Asthma control assessed according to GINA 2020 criteria (1), Asthma Control Test (2), or Asthma Control Questionnaire (3).

S-Figure 2: subject disposition

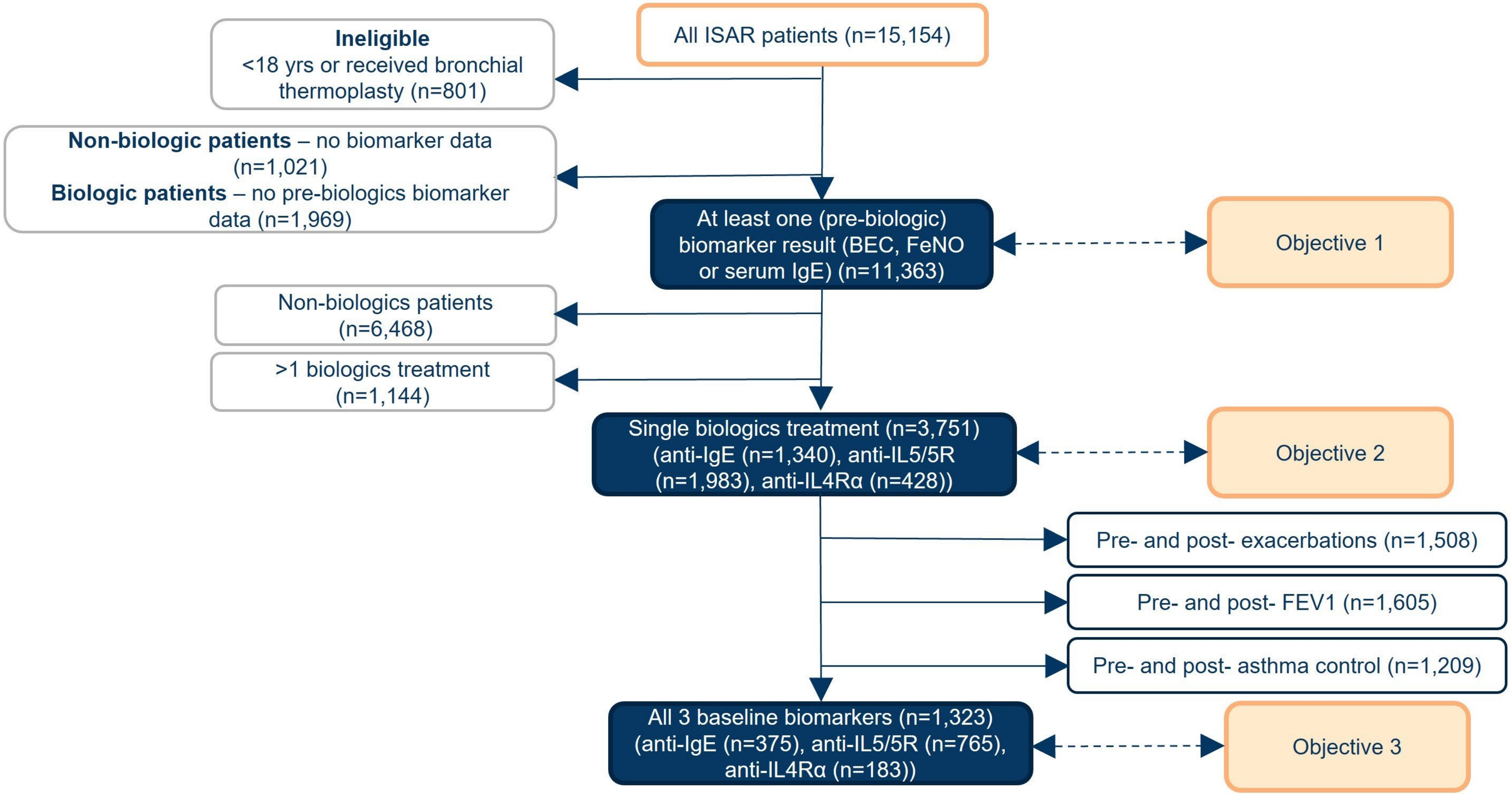

Abbreviations: Anti-IL4Rα, anti-interleukin 4 receptor alpha; anti-IL5/5R, anti-interleukin 5/5 receptor; BEC, blood eosinophil count; FEV_1, post-bronchodilator forced expiratory volume in one second; IgE, Immunoglobulin E; ISAR, International Severe Asthma Registry

Objective 1: Distribution of biomarkers and to assess whether biomarker concentrations correlate with each other.

Objective 2: To investigate if there is an association between pre-biologic biomarker concentrations and post-biologic asthma outcomes.

Objective 3: To identify whether multiple biomarker measurements lead to better prediction of biologic effectiveness.

Asthma control assessed according to GINA 2020 criteria (1), Asthma Control Test (2), or Asthma Control Questionnaire (3).

S-Figure 3: Distribution of pre-biologic biomarkers (highest concentration) among patients enrolled in ISAR for all patients and according to LTOCS use at baseline

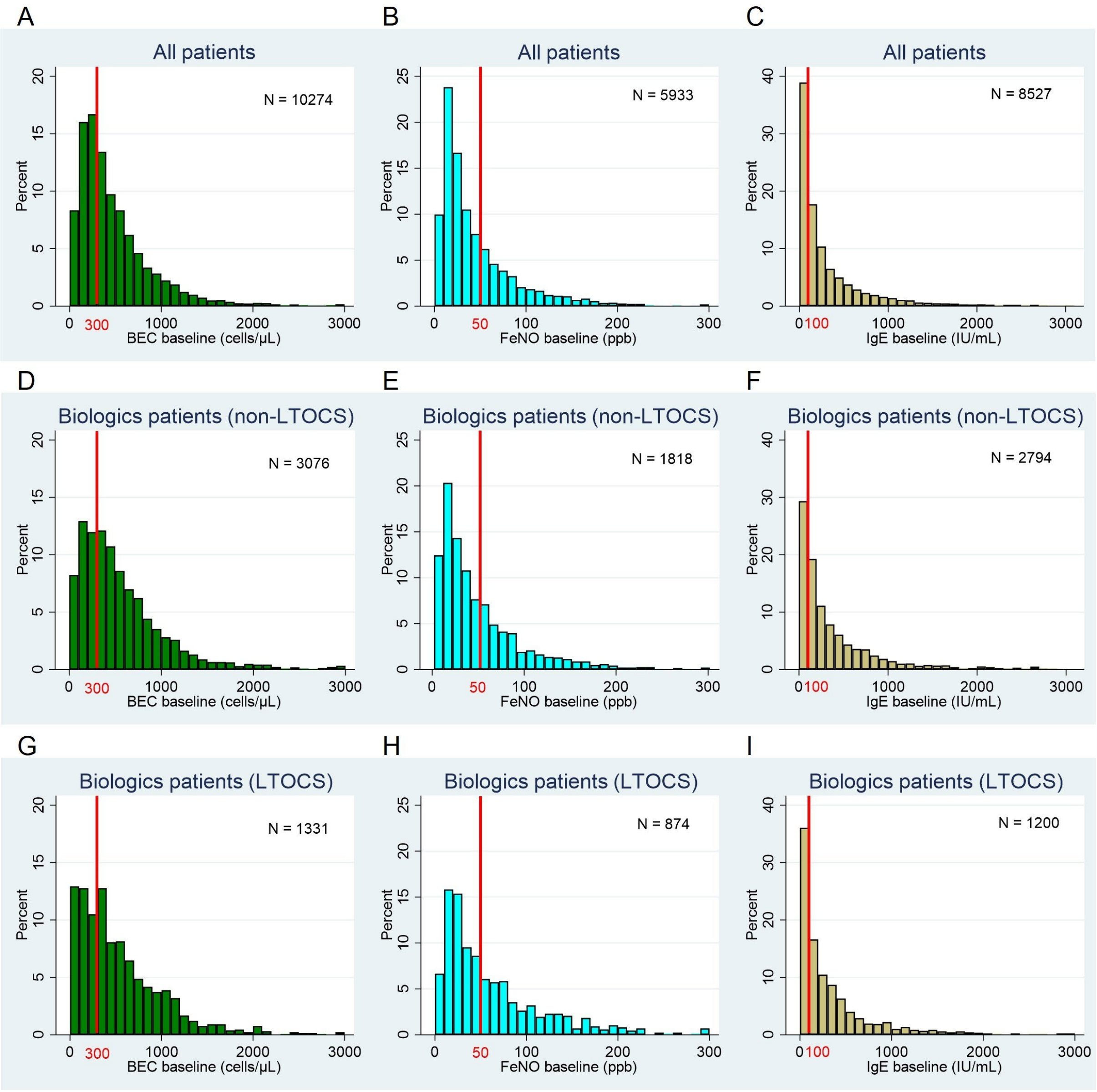

Abbreviations: BEC, blood eosinophil count; FeNO, fractional exhaled nitric oxide; IgE, immunoglobulin E; ISAR, International Severe Asthma Registry; ppb, parts per billion.

Selected clinically relevant cut-points are shown in red.

S-Figure 4: Median biomarker changes compared with baseline, at different times after initiation of biologic therapy

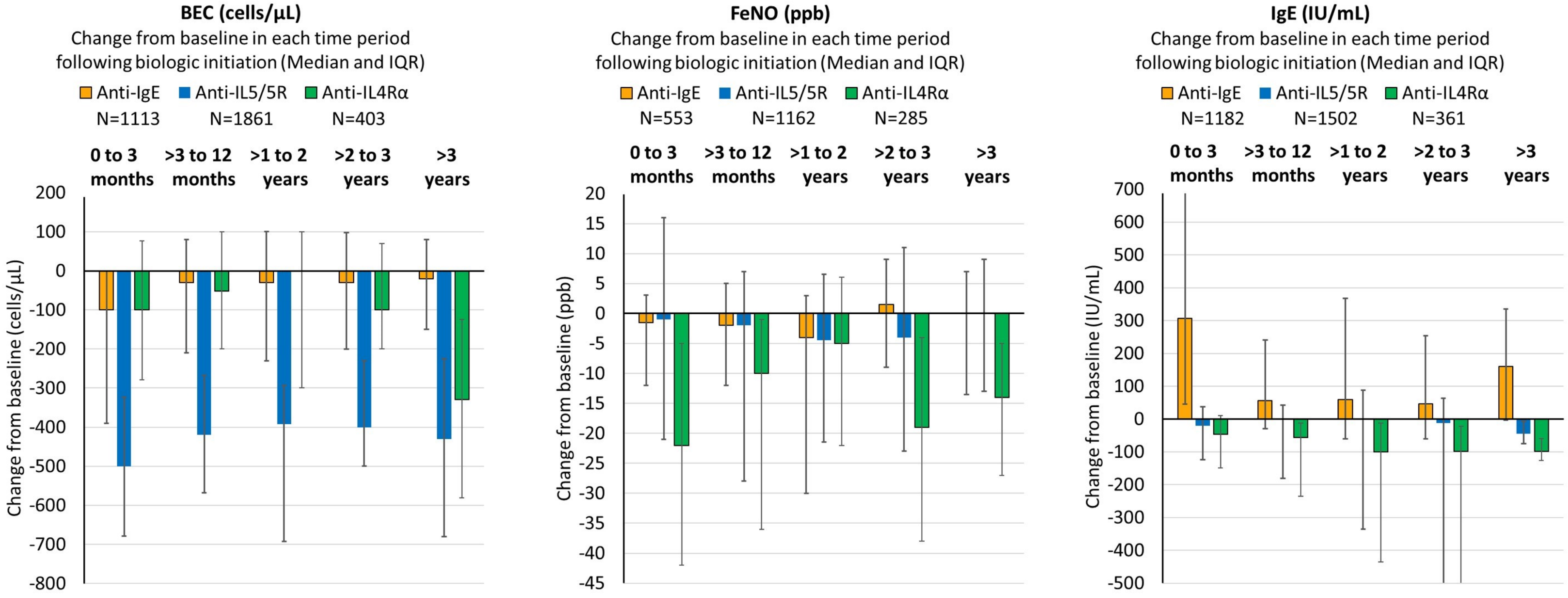

Abbreviations: Anti-IL4Rα, anti-interleukin 4 receptor alpha; IL5/5R: interleukin 5/5 receptor; BEC, blood eosinophil count; FeNO, fractional exhaled nitric oxide; IgE, immunoglobulin E; IQR, inter-quartile range

N numbers vary by time post-biologic initiation and are summarized in S-Table 2

S-Figure 5: Correlation of pre-biologic biomarkers taken within 7 days of each other among patients enrolled in ISAR

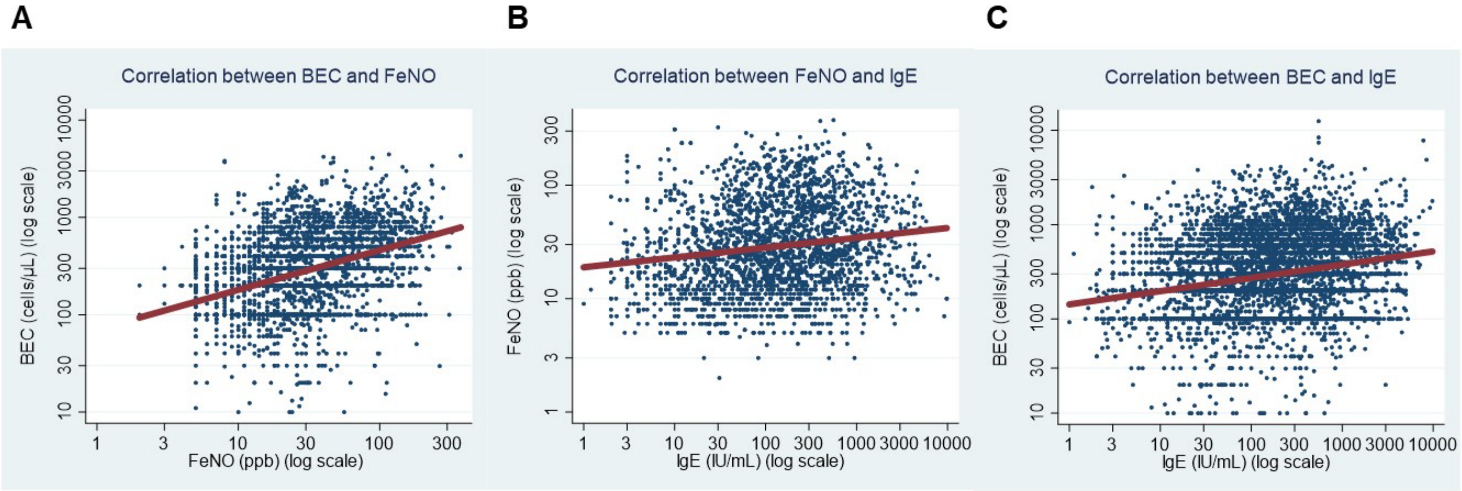

r = 0.40 (p<0.001)
N = 3099

r = 0.16 (p<0.001)
N = 2591

r = 0.25 (p<0.001)
N = 7144

Abbreviations: BEC, blood eosinophil count; FeNO, fractional exhaled nitric oxide; IgE, immunoglobulin E; ISAR, International Severe Asthma Registry; ppb, parts per billion; r: Pearson's correlation coefficient

S-Figure 6: Associations between post-biologic decrease in exacerbations and pre-biologic biomarker levels by subgroups

By presence or absence of allergies

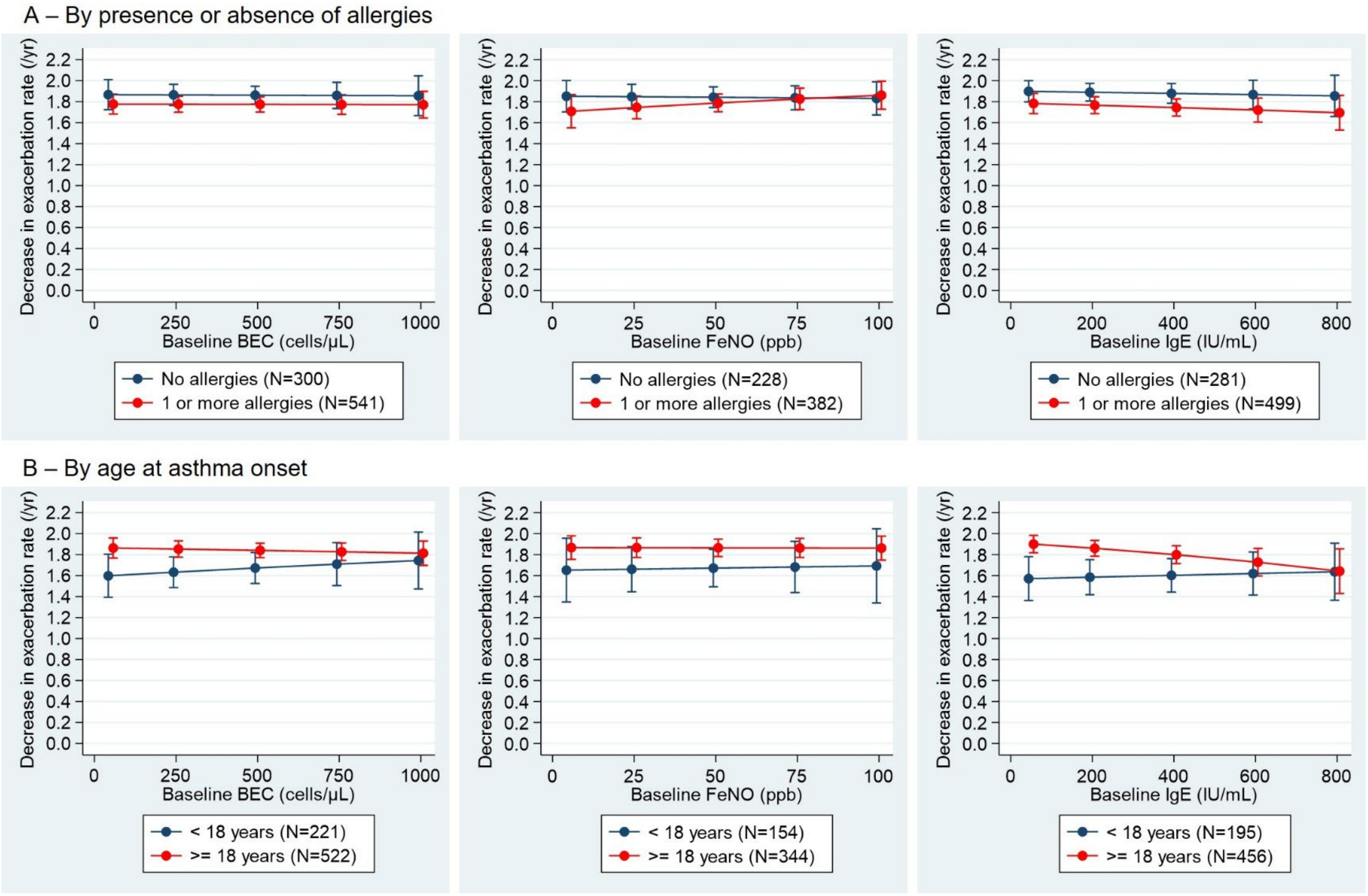

Abbreviations: BEC, blood eosinophil count; FeNO, fractional exhaled nitric oxide; IgE, immunoglobulin

S-Figure 7: Associations between post-biologic probability of uncontrolled asthma and pre-biologic biomarker levels by subgroups

(A) By baseline exacerbation rate

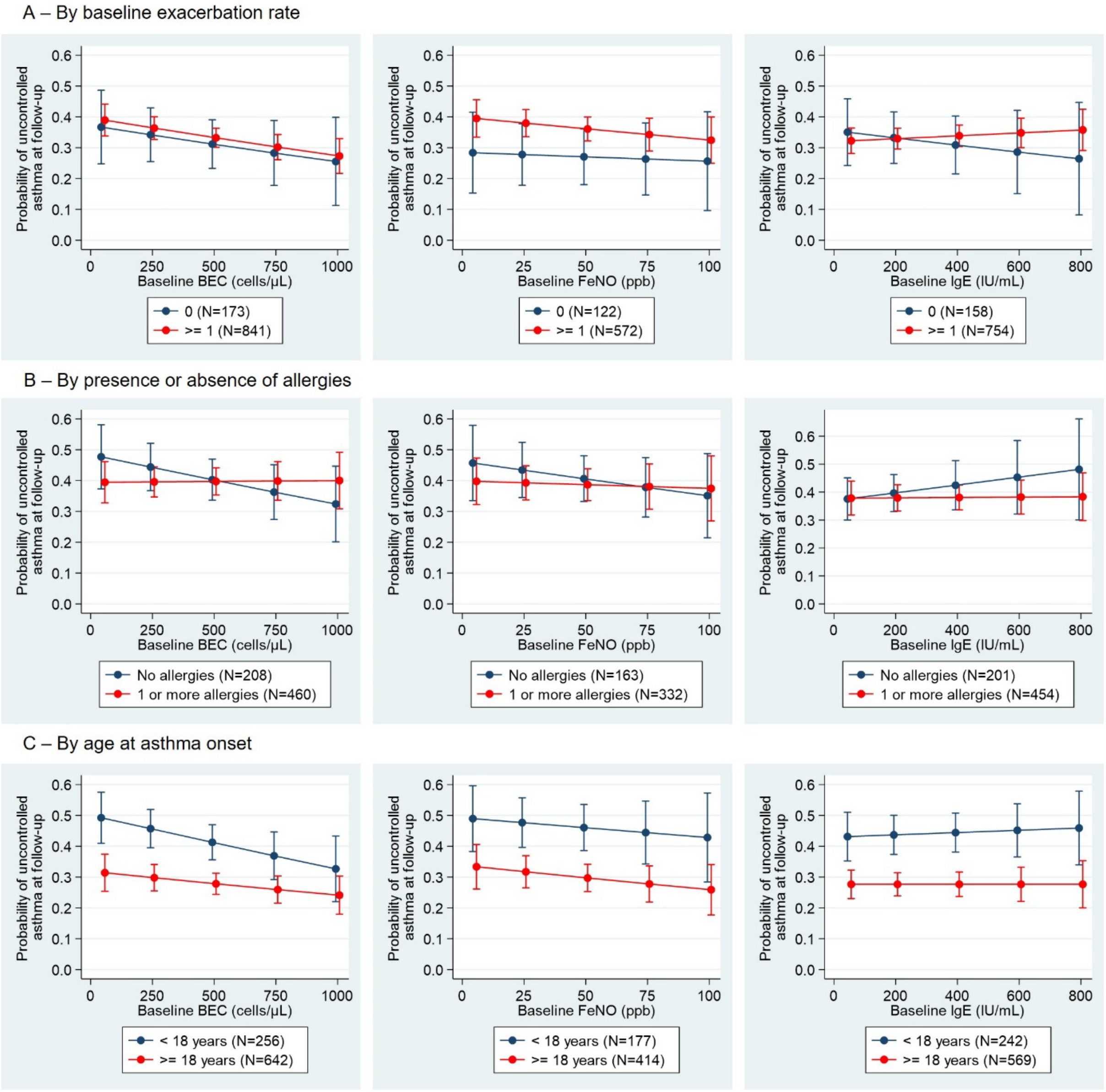

Abbreviations: BEC, blood eosinophil count; FeNO, fractional exhaled nitric oxide; IgE, immunoglobulin

Asthma control assessed according to GINA 2020 criteria (1), Asthma Control Test (2), or Asthma Control Questionnaire (3).

S-Figure 8: Associations between post-biologic improvement in FEV_1 and pre-biologic biomarker levels by subgroups

(A) By baseline exacerbation rate

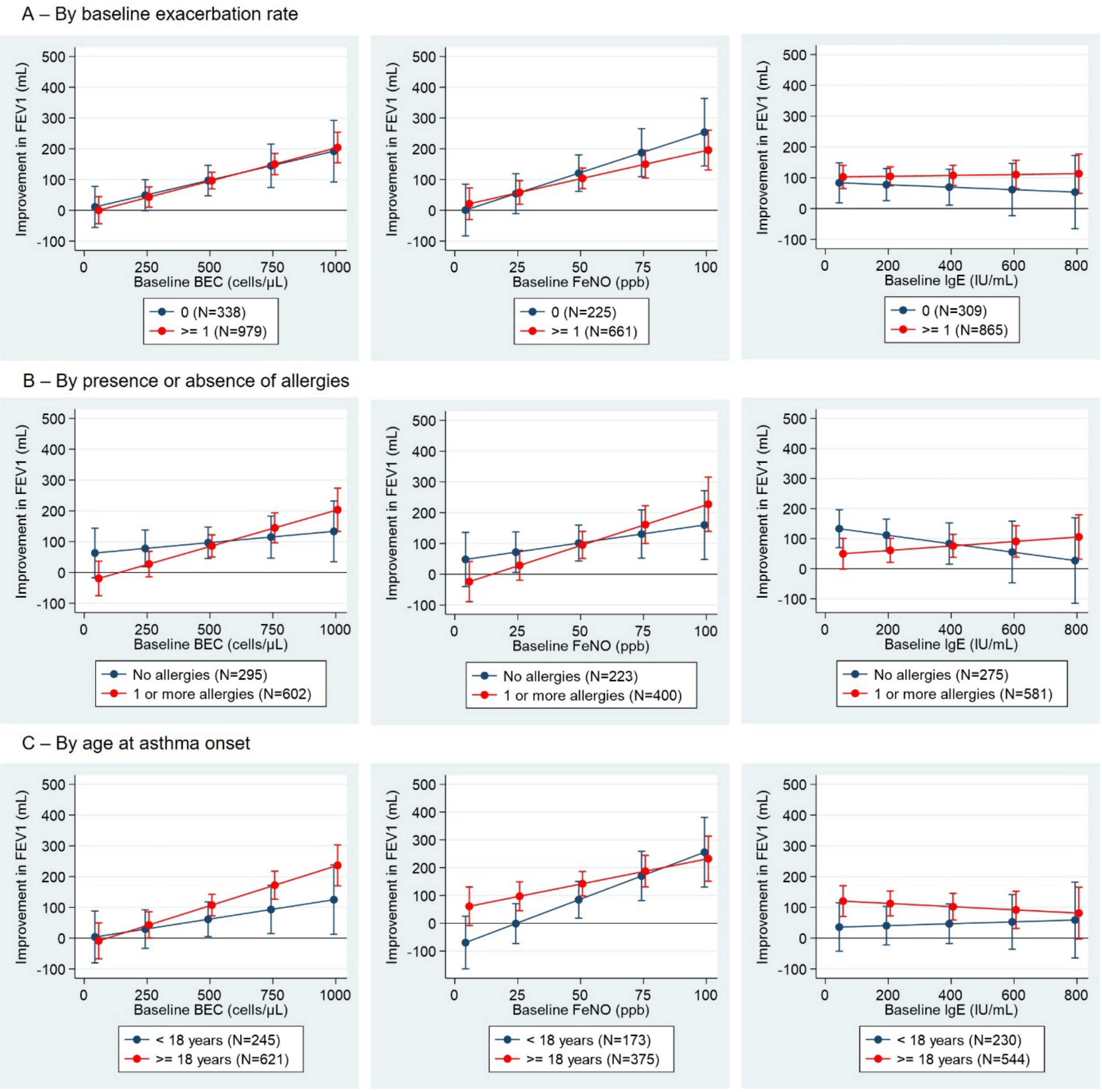

Abbreviations: BEC, blood eosinophil count; FeNO, fractional exhaled nitric oxide; FEV_1, forced expiratory volume in one second, IgE

S-Figure 9: Pre-to-post biologic change in FEV_1 (mL) according to BEC + FeNO categories

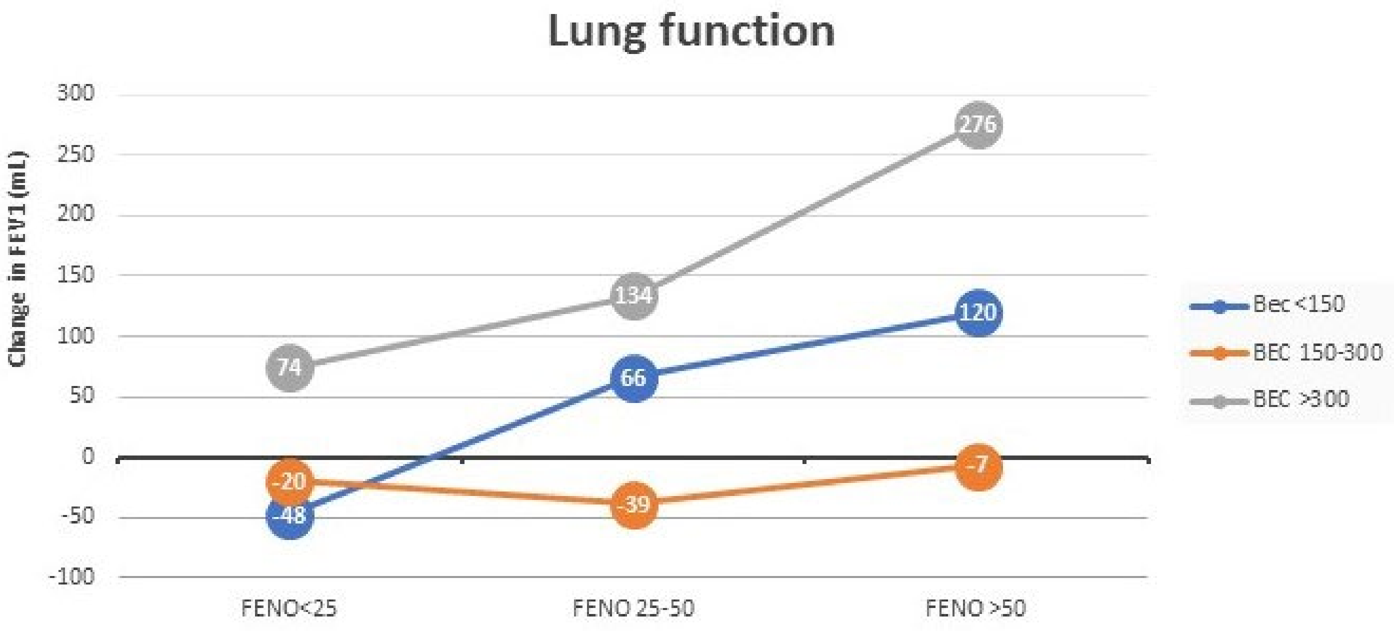

See **S-Table 8A** for n-numbers.

Abbreviations: BEC, blood eosinophil count (cells/µL); FeNO, fractional exhaled nitric oxide (ppb); FEV_1, forced expiratory volume in one second.

S-Figure 10: Association between baseline BEC and baseline exacerbation rate for patients, who (A) subsequently initiated and (B) did not initiate a biologic

A

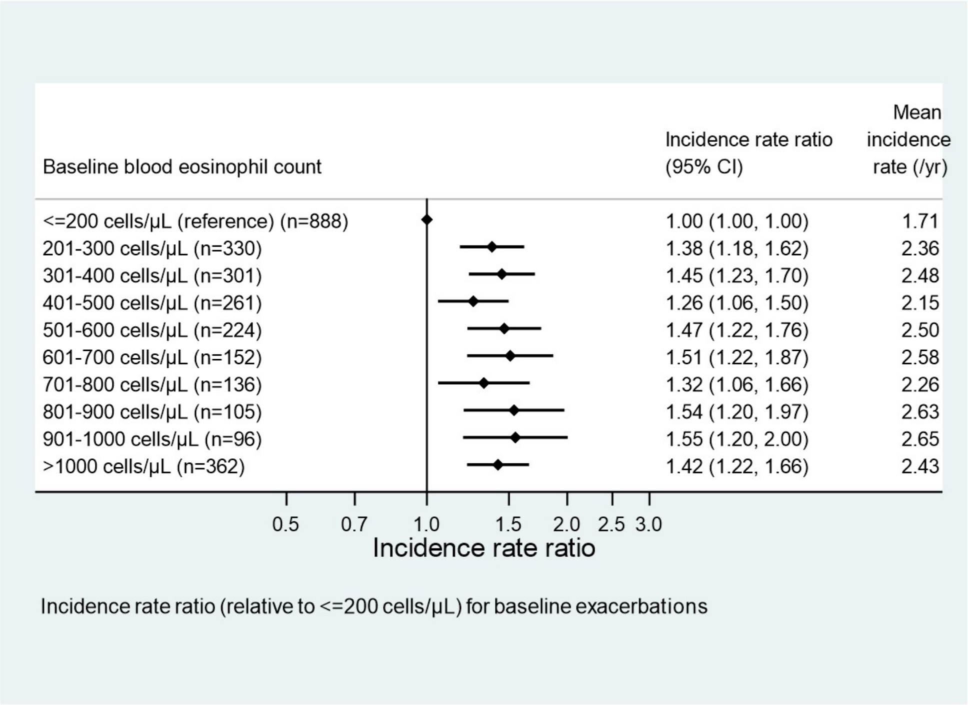

B

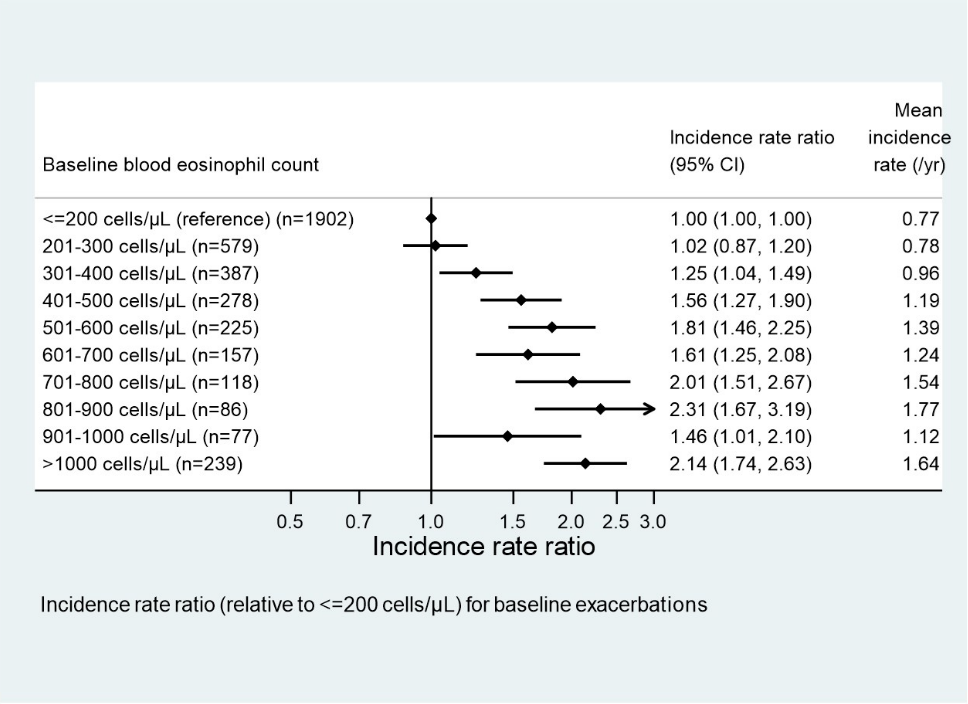

Abbreviations: BEC, blood eosinophil count; CI, confidence interval

References

1. Global Initiative for Asthma. Global Strategy for Asthma Management and Prevention. Updated 2020. https://ginasthma.org/wp-content/uploads/2020/04/GINA-2020-full-report_-final-_wms.pdf. [Last accessed 26th March 2024]

2. Nathan RA, Sorkness CA, Kosinski M, Schatz M, Li JT, Marcus P, Murray JJ, Pendergraft TB. Development of the asthma control test: a survey for assessing asthma control. J Allergy Clin Immunol (2004) 113:59–65. doi: 10.1016/j.jaci.2003.09.008

3. Juniper EF, O'Byrne PM, Guyatt GH, Ferrie PJ, King DR. Development and validation of a questionnaire to measure asthma control. Eur Respir J (1999) 14:902–907. doi: 10.1034/j.1399-3003.1999.14d29.x
